# Clear Cell Renal Cell Carcinoma Metastasis to the Thyroid: A Narrative Review of the Literature

**DOI:** 10.3390/cancers18010057

**Published:** 2025-12-24

**Authors:** Menelaos G. Samaras, Abraham Pouliakis, Konstantinos Skaretzos, Ioannis Boutas, Adamantia Kontogeorgi, Dionysios T. Dimas, Argyro-Ioanna Ieronimaki, Magda Zanelli, Andrea Palicelli, Maurizio Zizzo, Giuseppe Broggi, Rosario Caltabiano, Serena Salzano, Nektarios I. Koufopoulos

**Affiliations:** 1 Second Department of Pathology, Medical School, National and Kapodistrian University of Athens, Attikon University Hospital, 15772 Athens, Greece; apouliak@med.uoa.gr (A.P.); k.skarentzos@gmail.com (K.S.); anismed03@yahoo.gr (A.-I.I.); koufonektar@yahoo.com (N.I.K.); 2 Breast Unit, Rea Maternity Hospital, P. Faliro, 17564 Athens, Greece; drboutas@gmail.com; 3 Third Department of Obstetrics and Gynecology, Medical School, National and Kapodistrian University of Athens, Attikon University Hospital, 15772 Athens, Greece; ad.kontogewrgi@gmail.com; 4 Breast Unit, Athens Medical Center, Psychiko Clinic, 11525 Athens, Greece; dionysis.dimas@gmail.com; 5 Pathology Unit, Azienda USL-IRCCS di Reggio Emilia, 42123 Reggio Emilia, Italy; andreapalicelli@hotmail.it; 6 Surgical Oncology Unit, Azienda USL-IRCCS di Reggio Emilia, 42122 Reggio Emilia, Italy; maurizio.zizzo@ausl.re.it; 7 Department of Medical and Surgical Sciences and Advanced Technologies G.F. Ingrassia Anatomic Pathology, University of Catania, 95123 Catania, Italy; giuseppe.broggi@phd.unict.it (G.B.); rosario.caltabiano@unict.it (R.C.); 1000053265@studium.unict.it (S.S.)

**Keywords:** clear cell renal cell carcinoma, metastasis, thyroid

## Abstract

Clear cell renal cell carcinoma is the most common histological type of renal cancer, which is a common cancer type usually associated with a long clinical course. During this course, various metastatic sites can be observed. In this review, we have focused on metastases to the thyroid gland. We conducted research in three medical databases, including PubMed, Scopus, and Web of Science, using the same search algorithm. Our inclusion criteria focused on case reports and case series studies since 2011, covering therapeutic strategies for the primary and/or metastatic disease, as well as subsequent follow-up data. Studies with insufficient or uncertain data, or written in a language other than English, were excluded. An analysis of 510 articles from PubMed, 1729 from Scopus, and 649 from Web of Science, after application of inclusion and exclusion criteria, resulted in 77 reports, analyzing 189 patients. A description of the clinical, pathological, ancillary, and follow-up data, in the light of recent therapeutic schemes, was attempted. Our results suggest that metastases’ imaging features comprised more commonly a solitary nodule with a median size of 3.5 cm and worrisome features in ultrasonography, such as heterogeneity, hypoechogenicity, partially solid configuration, and variable internal vascularization. Histological and immunohistochemical examination of the lesion is necessary because these findings are not specific. Common non-thyroid metastases are seen in the urogenital system, lungs, and pancreas. We calculated the restricted mean survival from primary diagnosis at 274.6 months (95% CI: 264.3–285.0 months) and the restricted mean survival from thyroid metastases treatment at 93.9 months (95% CI: 65.3–122.4 months). Results regarding how patient characteristics affect these survival numbers were statistically nonsignificant (*p* > 0.05).

## Simple Summary

In this review, we have focused on the clinical, pathological, ancillary, and follow-up characteristics of clear cell renal cell carcinoma metastases to the thyroid gland. Data from 189 patients have been retrieved from 77 published case reports and case series. Since this is an unusual entity, this effort serves to organize the available information and introduce the novel therapeutic approaches used. We have compared our findings with those in already published reviews in this field. We have confirmed information regarding the clinical and diagnostic aspects of the disease, but we have calculated a higher restricted mean survival from primary diagnosis. We were unable to determine the impact of novel therapies on this finding, and we suggest that this poses a question for future research.

## 1. Introduction

Renal cancer (RC) is globally the sixth and ninth most common newly diagnosed cancer type in men and women, respectively [1]. The most common type of RC is clear cell renal cell carcinoma (ccRCC), which is observed mainly in patients over 60 years old in sporadic settings [2]. Clear cell renal cell carcinoma can also be associated with familial syndromes, including von Hippel–Lindau (VHL), constitutive chromosome 3 translocation, *BAP1* tumor predisposition, Cowden, Birt–Hogg–Dubé, and tuberous sclerosis. Several risk factors, among which are smoking, obesity, long-term dialysis, hypertension, diabetes mellitus, and exposure to toxic substances (asbestos, trichloroethylene), have been identified [3].

Clinically, ccRCC can be asymptomatic or present with hematuria, flank pain, and systematic symptoms when advanced. Metastatic sites can be heterogenous, may be detected years after the primary diagnosis, and may differ depending on which vascular branches are invaded (e.g., renal vein or lumbar veins). The lungs, central nervous system, head and neck structures, and bones are among the most frequent organ targets [4,5]. Metastatic disease appears to negatively impact patient prognosis, as the 5-year survival rate for stage IV patients is estimated at approximately 18%, whereas the 5-year survival rate for stage I patients is approximately 86% [2]. Targeted therapies constitute a promising treatment alternative to surgery for patients with advanced disease [6,7,8].

This review highlights the main diagnostic, novel therapeutic, and prognostic aspects of ccRCC metastases to the thyroid gland. While recent studies have addressed metastatic disease from various primaries to the thyroid [9,10,11], there are no recent [12] or thorough [13,14] reviews on metastatic disease from ccRCC to the thyroid. A relevant systematic review incorporated data published only until 2018 [15]. Our review adds to the current literature by analyzing numerous recent reports that describe 189 patients.

## 2. Materials and Methods

### 2.1. Study Design and Inclusion/Exclusion Criteria

The review was conducted according to the PRISMA (Preferred Reporting Items for Systematic reviews and Meta-Analyses) guidelines [16] and was not registered. The study selection criteria were defined by applying the PICO (Population/Participants, Intervention, Comparison, and Outcome) framework:

• Participants: Male or female patients regardless of age and race with metastatic ccRCC to the thyroid.
• Interventions: Any treatment intervention (surgery, chemotherapy, radiotherapy, immunotherapy, and targeted therapy) concerning primary ccRCC or thyroid metastasis was acceptable.
• Comparison: We were interested in pointing out the various different therapeutic approaches (novel and classical), rather than comparing them.
• Outcome: Interval from primary diagnosis of ccRCC to thyroid metastases and/or presence of disease relapse after treatment of thyroid metastases.

Original articles, including case series (studies reporting at least two cases) and single case reports, published from 2011 to March 2025, in peer-reviewed English-language journals, were deemed eligible for inclusion. Reports/series referring to at least three variables of interest (see Section 2.3) were included. Manuscripts written in languages other than English were excluded. Further exclusion criteria for the present review were as follows: (i) other article types: letters to the editor, comments, perspectives, guidelines, editorials, narrative or systematic reviews, and/or meta-analyses, (ii) papers available only as abstracts or with text appearing too brief or non-informative in terms of surgical and/or oncological data (e.g., image letters), and (iii) cases with an uncertain/doubtful diagnosis or other diagnosis than ccRCC.

### 2.2. Literature Search Strategy

Eligible studies were identified by searching through the MEDLINE (via PubMed), Scopus, and Web of Science databases (end-of-search date: 10 March 2025) by two independent reviewers. The following search algorithm was used: (renal cell carcinoma) AND (thyroid) AND (metastasis). Any disagreements on article inclusion were resolved by a third reviewer when necessary. The reference lists of the included studies were also thoroughly searched to identify eligible studies missed, using the “snowball” methodology [17].

### 2.3. Data Tabulation and Extraction

Data tabulation and extraction from the eligible articles were performed using a standardized, pre-piloted form. Two independent reviewers, blinded to each other, extracted the data, and any disagreements were resolved by a third reviewer when necessary. The following data were collected: (i) study characteristics (first author, year of publication, number of patients enrolled), (ii) clinical and ancillary parameters (sex, age, clinical symptoms, physical examination findings, thyroid hormones status, imaging studies, synchronous neoplasia, non-thyroid metastases prior or synchronous, metastatic disease treatment, follow-up after thyroid metastasis, recurrence, interval to recurrence, and outcome), (iii) thyroid metastases features (dimensions, number, laterality, immunophenotype), and (iv) history of ccRCC (clinical stage, grade, kidney disease treatment, interval to thyroid metastasis).

### 2.4. Tools for Data Processing and Statistical Analysis

The statistical analysis was performed using the R language version 4.5.1 [18]. Descriptive characteristics of the quantitative data were reported as median (range) and, for completeness, as mean ± standard deviation (SD). For the qualitative data, the frequency of occurrence and the relevant percentage were reported. Follow-up period and patient status at follow-up end were not reported for all patients; however, numerous cases reported the interval to metastasis. For these patients, the follow-up period was defined as the interval to metastasis, and they were considered alive for survival analysis. Survival curves were constructed using the Kaplan–Meier method, and the restricted mean survival time (RMST) was calculated using the Greenwood approach. Finally, the potential role of the recorded characteristics in overall survival (OS) was evaluated using the log-rank method.

## 3. Results

### 3.1. Study Selection

Our search retrieved 2888 unique articles, of which 118 underwent full-text evaluation for eligibility. Finally, 77 reports, 79% of which were published since 2015, were eligible for our review. Whenever authors referred to RCC in their diagnosis, we assumed that the histopathologic equivalent was the most common type, namely ccRCC, and thus included those articles in our review. We excluded articles that did not provide detailed information about individual patients. A PRISMA flow diagram summarizing the search results is shown in Figure S1. The baseline characteristics of the patients are shown in Table 1.

### 3.2. Demographic, Clinical, and Imaging Features

The analyzed reports described 189 patients with a median age of 65 (range: 39–88) years (calculated from the available data for 189 patients). A total of 97 (97/185, 52%) patients were male, and 88 (88/185, 48%) patients were female (4 non-applicable).

The main presenting symptoms were reported in 60/77 manuscripts [13,14,19,20,21,22,23,24,25,26,27,28,29,30,31,32,33,34,35,36,37,38,39,40,41,42,43,44,45,46,47,48,49,50,51,52,53,54,55,56,57,58,59,60,61,62,63,64,65,66,67,68,69,70,71,72,73,74,75,76]. A total of 24 (24/120, 20%) patients were asymptomatic, while 96 (96/120, 80%) patients presented with the following symptomatology: 60 (60/96, 63%) patients with a neck mass, 7 (7/60, 12%) of whom had compressive symptomatology (dysphagia, dyspnea, neck pain, cough), and 2 (2/60, 3%) of whom had systematic symptomatology (weight loss), 16 (16/96, 17%) patients mainly with compressive symptomatology (dysphagia, dyspnea, pain/discomfort, voice hoarseness, cough), 1 (1/16, 6%) of whom had systematic symptomatology (fatigue, loss of appetite), 17 (17/96, 17%) patients with goiter/neck enlargement/thyroid enlargement/thyroid lobe enlargement/thyroid nodule size increase, 5 (5/17, 29%) of whom had compressive symptomatology (dyspnea, neck pain, voice hoarseness), 2 (2/96, 2%) patients with symptomatology from non-thyroid metastases, and 1 (1/96, 1%) patient with preauricular mass (69 non-applicable).

Physical examination findings were reported in 47/77 manuscripts [13,14,19,21,22,23,26,27,28,29,30,31,34,35,36,37,38,39,40,42,43,45,46,47,48,49,50,51,52,53,54,57,58,59,60,61,62,63,66,68,69,70,73,74,75,76,77]. Physical examination revealed a palpable thyroid gland, thyroid/neck mass, or thyroid nodules in 43 (43/53, 81%) patients, non-thyroid metastases in 1 (1/53, 2%) patient, and in 9 (9/53, 17%) patients, it was unremarkable (136 non-applicable). The palpable fulness was indurated in 11 (11/20, 55%) patients and painless in 9 (9/20, 45%, 23 non-applicable).

Referral to imaging studies was applicable in 62/77 manuscripts [13,14,19,20,21,22,23,24,25,26,27,28,29,31,32,33,34,35,36,37,38,39,40,41,42,43,44,45,46,47,48,49,50,51,52,53,54,57,58,59,60,62,63,66,67,68,69,70,71,72,73,74,75,76,77,78,79,80,81,82,83,84]. To detect metastases, 44 (44/79, 56%) patients underwent ultrasonography (U/S) only, 10 (10/79, 13%) patients U/S and computed tomography (CT), 8 (8/79, 10%) patients CT only, 5 (5/79, 6%) patients positron emission tomography (PET/CT) only, 4 (4/79, 5%) patients U/S and PET/CT, 2 (2/79, 3%) patients U/S and scintigraphy, 2 (2/79, 3%) patients magnetic resonance imaging (MRI) and PET/CT, 1 (1/79, 1%) patient U/S and MRI, 1 (1/79, 1%) patient CT and scintigraphy, 1 (1/79, 1%) patient scintigraphy only, and 1 (1/79, 1%) patient endoscopic ultrasound (110 non-applicable). Due to the variability of imaging findings, a practical grouping was not considered feasible. An analytical presentation for each report is provided in Table S3.

Synchronous neoplasia was discovered in 16/77 manuscripts [20,37,40,47,48,50,51,52,53,66,68,72,73,76,79,84] and in 16 patients (16/81, 20%, 108 non-applicable). Six (6/16, 38%) patients suffered from papillary thyroid carcinoma, two (2/16, 13%) patients from esophageal adenocarcinoma, two (2/16, 13%) patients from follicular adenoma, one (1/16, 6%) patient from follicular and one (1/16, 6%) patient from papillary thyroid microcarcinoma, one (1/16, 6%) patient from hyalinizing trabecular tumor of the thyroid, one (1/16, 6%) patient from microinvasive HÜrthle cell carcinoma, one (1/16, 6%) patient from pancreatic neuroendocrine neoplasm, and one (1/16, 6%) patient from metastatic breast carcinoma.

Thyroid hormones were examined in 30/77 manuscripts [14,25,27,29,32,33,34,35,38,41,42,43,44,46,49,50,52,54,59,60,62,63,66,68,69,70,73,74,75,76]. In terms of thyroid hormone status, 27 (27/33, 82%) patients were euthyroid, 3 (3/33, 9%) patients had subclinical hypothyroidism, 1 (1/33, 3%) patient hyperthyroidism, 1 (1/33, 3%) patient elevated free T4 (fT4), and 1 (1/33, 3%) patient normal thyrotropin stimulating hormone (TSH) and free T3 (fT3) and decreased fT4 (156 non-applicable). An analytical presentation for each report is provided in Table S1.

### 3.3. History of ccRCC

Staging and grading data were reported in 39/77 manuscripts [13,19,22,23,25,26,29,30,31,34,35,36,37,38,40,44,46,47,48,49,52,54,56,57,58,59,60,61,62,63,66,68,69,70,73,75,80,82,84,85]. Totals of 20 (20/44, 45%), 5 (5/44, 11%), 12 (12/44, 27%), and 7 (7/44, 17%) patients were diagnosed with stage I, II, III, and IV ccRCC, respectively (145 non-applicable). The eighth version of the American Joint Committee on Cancer (AJCC) Tumor, Node and Metastasis (TNM) staging system was used to match the various pathology TNMs (pTNM) to their equivalent stage [86]. Regarding the World Health Organisation/International Society of Urologic Pathology (WHO/ISUP) or Fuhrman grading systems, ccRCC was grade 1, 2, 3, and 4 for 4 (4/41, 10%), 20 (20/41, 49%), 14 (14/41, 34%), and 3 (3/41, 7%) patients, respectively (148 non-applicable). A total of 32 (32/61, 52%) patients had right-sided and 29 (29/61, 48%) patients had left-sided ccRCC (128 non-applicable).

Therapy of primary kidney disease was documented in 61/77 manuscripts [13,14,19,20,21,22,23,24,25,26,27,28,29,30,31,32,33,34,35,36,37,38,39,40,41,42,43,45,46,47,48,49,50,52,53,54,56,57,58,59,60,61,62,63,66,68,69,70,72,73,74,75,76,77,78,79,80,82,84,85,87]. A total of 3 (3/71, 4%) patients underwent partial nephrectomy, 37 (37/71, 52%) patients total nephrectomy, 30 (30/71, 42%) patients radical nephrectomy, and 1 (1/71, 2%) patient total nephrectomy and contralateral adrenalectomy (118 non-applicable). Nine (9/20, 45%) stage I patients underwent total nephrectomy, eight (8/20, 40%) radical, and three (3/20, 15%) partial. Three (3/5, 60%) stage II patients underwent radical nephrectomy, and two (2/5, 40%) total. Eight (8/12, 67%) stage III patients underwent radical nephrectomy, and four (4/12, 33%) total. Finally, four (4/7, 57%) stage IV patients underwent radical nephrectomy, two (2/7, 29%) total, and one (1/7, 14%) received total, and contralateral adrenalectomy.

Non-thyroid metastases history was reported in 22/77 manuscripts [13,14,19,21,22,23,24,25,26,37,45,47,48,49,51,73,74,77,78,79,80,84]. Fifteen (15/80, 19%) patients had synchronous-to-thyroid metastases (109 non-applicable). Non-thyroid metastatic sites were the lungs/mediastinum for four (4/15, 26,66%) patients, the contralateral kidney for one (1/15, 6,66%) patient, the pancreas for one (1/15, 6,66%) patient, distal lymph nodes (upper abdominal) for one (1/15, 6,66%) patient, the head and neck area for one patient (1/15, 6,66%), and the central nervous system for one (1/15, 6,66%) patient, while six (6/15, 40%) patients presented with multiple metastases (lung/mediastinum, adrenals, pancreas, forearm, contralateral kidney, mesenteric lymph nodes, urogenital system, skeleton, retroperitoneum).

Sixteen (16/80, 20%) patients had prior-to-thyroid metastases (109 non-applicable). Non-thyroid metastatic sites were the urogenital system, adrenal glands, and retroperitoneum for four (4/10, 40%) patients, the pancreas for three (3/10, 30%) patients, the lungs/mediastinum for one patient (1/10, 10%), and distal lymph nodes (para-aortic) for one (1/10, 10%) patient, while one (1/10, 10%) patient presented with multiple metastases (pancreas, adrenals, lung) (six non-applicable).

Features of thyroid metastases were documented in 72/77 manuscripts [13,14,19,20,21,22,23,24,25,26,28,29,30,31,32,33,34,35,36,37,38,39,40,41,42,43,44,45,46,47,48,49,50,51,52,53,54,55,56,57,58,59,60,62,63,64,65,66,67,68,69,70,71,72,73,74,75,76,77,78,79,80,81,82,83,84,87,88,89,90,91,92]. Solitary metastasis from ccRCC to the thyroid gland was present in 97 (97/126, 77%) patients. In contrast, multiple metastases were detected in 29 (29/126, 23%) patients (63 non-applicable), ranging from 2 to 6. A total of 47 (47/113, 42%) metastases occupied the right thyroid lobe, 38 (38/113, 34%) the left thyroid lobe, 26 (26/113, 23%) both lobes, and 1 (1/113, 1%) metastasis occupied the left thyroid lobe and isthmus and the thyroid isthmus only, respectively (76 non-applicable). The median size of their largest dimension was 3.5 (range: 0.8–10) centimeters (cm). An analytical presentation for each report is provided in Table S2.

Histologically, the most prevalent metastatic growth pattern was a nested pattern, observed in 16 (16/24, 67%) patients (165 non-applicable). Solid, glandular, and trabecular patterns were observed to a lesser extent. The characteristic clear/glycogen abundant cytoplasm and the delicate vascular network were recorded in 35 (35/35, 100%) (153 non-applicable) and 18 (18/18, 100%) (171 non-applicable) patients, respectively. For 60 patients, immunohistochemistry was performed with some of the following markers: epithelial membrane antigen (EMA), which confirms epithelial origin, paired box gene 8 (PAX8), which confirms renal origin, cluster of differentiation 10 (CD10), vimentin, renal cell carcinoma marker (RCCm), and carbonic anhydrase 9 (CAIX), which are used to differentiate ccRCC from other variants. An analytical presentation for each report is provided in Table S4.

### 3.4. Treatment Outcome

Treatment of metastatic disease and/or patient outcome was reported in 70/77 manuscripts [13,14,19,20,21,22,23,24,25,26,27,28,29,30,31,32,33,34,35,36,37,38,39,40,41,42,43,44,46,47,48,49,50,51,52,53,55,56,57,58,59,60,61,62,63,66,67,68,69,70,71,72,73,74,75,76,77,78,79,80,81,82,83,84,85,87,88,89,90,91,92,93,94].

Various therapeutic approaches were employed. A total of 47 (47/143, 33%) patients underwent thyroid lobectomy/hemithyroidectomy, 2 (2/47, 4%) of them with lymph node dissection. Three (3/47, 6%) patients received adjuvant therapy including immunotherapy (non-applicable scheme), chemotherapy (non-applicable scheme), and tyrosine kinase inhibitors (TKIs) (sunitinib). A total of 73 (73/143, 51%) patients underwent total thyroidectomy. Ten (10/73, 14%) patients received adjuvant therapy including radiotherapy, chemotherapy (non-applicable scheme), immunotherapy (non-applicable scheme), TKIs (sunitinib, pazopanib), mechanistic target of rapamycin (mTOR) inhibitors (temsirolimus), and interferon a (INFa). Seven (7/143, 5%) patients underwent radical thyroidectomy. Two (2/7, 29%) patients received adjuvant therapy including INFa and TKIs (sunitinib). Radiotherapy and/or systematic therapy, including chemotherapy (non-applicable scheme), targeted therapy [TKIs (sunitinib, pazopanib), mTOR inhibitors (temsirolimus), kinase inhibitors (sorafenib)], and immunotherapy (nivolumab) without surgical intervention were used in 10 (10/143, 7%) patients. Other therapeutic strategies were documented in six (6/143, 4%) patients (46 non-applicable).

Overall, 26 (26/119, 22%) patients were reported to be alive without further clarification of their disease status; 10 (10/119, 8%) patients were alive with disease; and 44 (44/119, 37%) patients were alive without disease. A total of 26 (26/119, 22%) patients died of unknown causes, whereas 13 (13/119, 11%) patients died of their disease (70 non-applicable). A total of 87 (87/114, 76%) patients had undergone surgical (total/radical thyroidectomy, hemithyroidectomy) therapy, 15 (15/114, 13%) patients surgical (total/radical thyroidectomy, hemithyroidectomy) therapy with adjuvant (TKI, INFa, ICI, mTOR inhibitors, chemotherapy, radiotherapy) treatment, 8 (8/114, 7%) patients received systematic treatment, and 4 (4/114, 4%) patients received other therapy (5 non-applicable).

Median follow-up was 24 (range: 1,5–156) months (calculated based on the available data from 51 patients). A total of 11 (11/50, 22%) patients were reported to be alive in the last follow-up without further clarification of their disease status, 5 (5/50, 10%) patients were alive with disease, while 23 (23/50, 46%) patients were alive without disease. Eight (8/50, 16%) patients died of unknown causes, whereas three (3/50, 6%) patients died of their disease (1 non-applicable). A total of 37 (37/50, 74%) patients had undergone surgical (total/radical thyroidectomy, hemithyroidectomy) therapy, 10 (10/50, 20%) patients surgical (total/radical thyroidectomy, hemithyroidectomy) therapy with adjuvant (TKI, INFa, ICI, chemotherapy, radiotherapy) treatment, 2 (2/50, 4%) patients received TKI/ICI, and 1 (1/50, 2%) patient received palliative therapy (1 non-applicable).

Recurrence was documented in 11 (11/43, 26%) patients (146 non-applicable). The first recurrence occurred at 12 months in four (4/11, 36%) patients, at 24 months in two (2/11, 18%) patients and at 1, 6, 36, 48, and 72 months in one (1/11, 9,2%) patient, respectively. Recurrence sites were multiple for most patients and frequently included the lungs, pancreas, central nervous system, forearm, and contralateral kidney. An analytical presentation for each report is provided in Table S1.

From the available reports, only those providing adequate information were included in the survival analysis. Survival outcome with initiation points of the time of diagnosis were available for 179 patients (note that when the outcome was not reported but instead a metastatic disease was reported, then these patients were considered alive up to the time point of reported metastasis). However, time-related data were available for 150 patients; from these data, 23 described death events and the remaining patients were alive up to the follow-up point. Additionally, time information since metastasis was available for 51 patients, and thus it was possible to create survival curves with the initiation point of the time of metastasis; from these 51 patients, 11 were reported as dead and the remaining patients were reported as alive. Due to the limited information on those still alive, death from any reason was considered as a terminal event.

**Figure 1:**
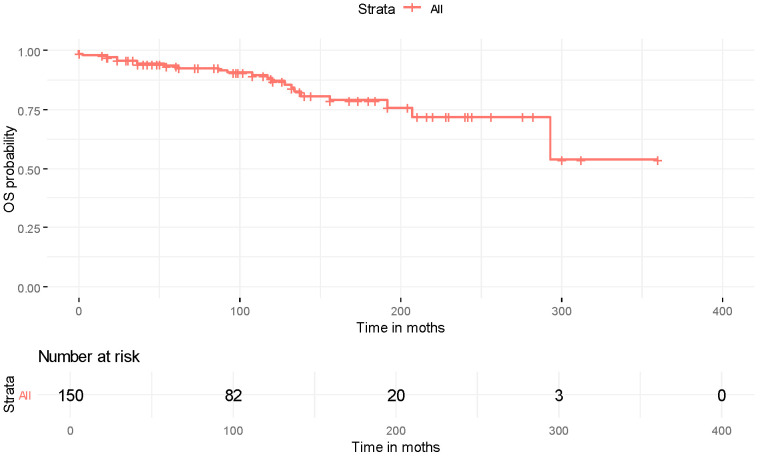
Kaplan–Meier curve for overall survival after diagnosis along with patients at risk.

**Figure 2:**
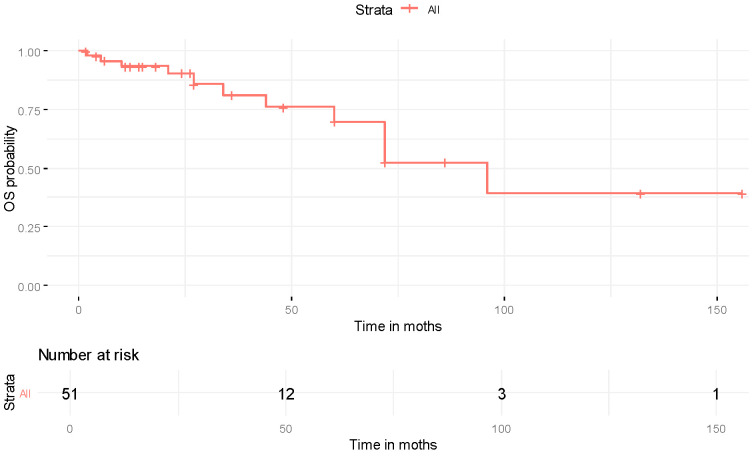
Kaplan–Meier curve for overall survival after metastasis along with patients at risk.

The survival curve (starting from diagnosis) along with patients at risk is presented in Figure 1. The RMST was 274.6 months (95% CI: 264.3–285.0 months). The potential role of the recorded characteristics in the OS was evaluated, but did not yield statistically significant results, and is presented in Table S5. Supporting diagrams of Table S5 are presented in Figure S2. The RMST for survival after thyroid metastases was 93.9 months (95% CI: 65.3–122.4 months), while the median survival time was 96 months. The survival curve and the patients at risk are presented in Figure 2. The evaluation of study characteristics in survival after thyroid metastases did not produce statistically significant results and is presented in Table S6.

Despite the high percentage of missing data, we have performed Cox proportional hazards regression analysis to investigate numerous other patient characteristics (based on the available data) such as the following: gender (146 cases), other synchronous neoplasia (69 cases), prior non-thyroid metastasis (68 cases), synchronous non-thyroid metastasis (68 cases), single or multiple solitarity (107 cases), laterality (R: right, L: left, B: both lobes, 95 cases), stage (38 cases), and grade (40 cases); none of these characteristics was found to have a role in patient survival.

## 4. Discussion

In this review, we have addressed the epidemiologic, diagnostic, and therapeutic aspects of ccRCC metastases to the thyroid gland. This effort serves as an update to the current literature, encompassing data from case reports and case series published within the last 15 years, thus providing valuable insights into novel diagnostic and therapeutic strategies in the field.

The clinical course of ccRCC is long, compared to other cancer types; however, it depends on clinical stage [2,3]. It is therefore reasonable to observe late, distant metastases to various organs [5]. While the thyroid is perfused by a well-developed vascular network, clinically relevant metastases are rare [95]. Microscopic metastases are more common, as demonstrated in autopsy studies [4]. The patients we analyzed (M:F ratio = 1.1) presented, in majority, with noticeable clinical symptomatology. The main complaint was a neck mass that, when large enough, compressed adjacent structures. Physical examination revealed a palpable mass in most patients. Similar findings are reported in the literature, but are not specific for metastatic ccRCC [12]. The ancillary imaging studies performed were variable, with a preference for plain ultrasonography. Sonographic characteristics included a mainly heterogenous, hypoechoic, partially solid nodule with variable internal vascularization and thrombosis of the adjacent veins. These findings are confirmed by another review [12]. During the imaging work-up, the presence of a second malignancy was identified in a few patients, mainly papillary thyroid carcinoma and, surprisingly, esophageal adenocarcinoma. Only a few of the reports we analyzed referred to the patients’ thyroid hormone status. However, the available data indicated that metastatic ccRCC did not affect thyroid function, as most patients were tested euthyroid, which is also reported in a retrospective study [96].

Regarding the history of ccRCC, most patients suffered from stage I disease at the time of initial diagnosis. This finding contrasts with other case series, in which most of the patients suffered from stage II or III disease [97,98]. A possible explanation for the metastatic behavior of ccRCC in lower clinical stage cases is high histological grade, since it is known that ccRCC grading affects patient prognosis [99]. Following classical treatment proposals [100], most patients with localized disease underwent total or radical nephrectomy. Patients with metastatic disease at the time of the primary diagnosis underwent total or radical nephrectomy with or without adrenalectomy; however, they did not receive adjuvant therapy. According to recent treatment guidelines for metastatic ccRCC, patients may undergo active surveillance, cytoreductive surgery, or receive a combination of immune checkpoint inhibitors (ICIs) with or without a vascular endothelial growth factor receptor tyrosine kinase inhibitor (VEGFR TKI), depending on clinical criteria [101]. The most common metastatic sites in our patients, whether prior or synchronous to thyroid, were the lungs and mediastinum, the urogenital system, and the pancreas. The metastatic pattern could be solitary or multiple. A relationship between thyroid and pancreatic metastases from ccRCC has been implied before [97,98].

Solitary metastasis to the thyroid was more frequent than multiple metastases, with a median size of 3,5 cm. The prevalence of solitary metastasis with a median size of 3,5 cm is referred to elsewhere in the literature [8]. The characteristic histopathological features of the primary site, namely a nested pattern with clear cells surrounded by a delicate vascular network, are retained in many of our patients and in the literature [96]. PAX8 and CAIX are important immunohistochemical markers to differentiate ccRCC from primary thyroid mimicker neoplasms [102]. Apart from those markers, CD10, RCCm, and EMA were also expressed in most of our patients.

The treatment of metastatic thyroid disease from ccRCC has been surgical for many years, judging by the numerous single-/multi-center studies in this field, and consists of some thyroidectomy (total, radical, or partial) or lobectomy with or without lymph node dissection, depending on the anatomical distribution and extent of metastatic disease [8,97,98,103,104]. It is stated that overall survival is unaffected by the type of surgical treatment, when total, subtotal, or palliative thyroidectomy is performed [97,98]. Even though total thyroidectomy has been suggested to prevent local recurrences in the head and neck area compared with more conservative approaches [97], this has not been proven in case series [8,96,105]. In our review, we have included patients who received adjuvant therapy after surgery, those who received surgery alone, and those who received only systemic treatment. In addition to chemotherapy and radiotherapy, among new therapeutic agents are the TKIs sunitinib and pazopanib, the mTOR inhibitor temsirolimus, IFNa, the kinase inhibitor sorafenib, and the ICI nivolumab. These targeted therapies are generally within the available therapeutic options for metastatic ccRCC [6,7,100,101]. Our results indicate that OS from diagnosis of primary kidney disease is 274.6 months, significantly higher than 147.6 and 199.2 reported in single-center studies with 36 and 34 patients treated with only surgery or palliative therapy [96,98]. Regarding survival after treatment for metastatic thyroid disease, in a median follow-up time of 24 months, 78% of our patients were alive with or without disease. A total of 24% of them had received adjuvant post-surgery systematic therapy or systematic therapy as monotherapy. RMST was 93.9 months, which falls between 70.8 [98], 71 [97], 76.8 [96], and 100 [8] months, reported survival rates after treatment in other studies. Risk factors affecting OS from initial diagnosis and survival after metastases treatment include metastases to the contralateral kidney or other nonendocrine organs, more than four thyroid metastases, spread to adjacent cervical structures, age >= 70 years, and palliative surgery [8,97].

Our calculations of RMST regarding overall survival and survival after thyroid metastases are higher than the calculated survival rates in the literature cited above. Several reasons, particularly methodological and clinical, might explain this finding. We have calculated RMST, while other authors have calculated median or mean values. This difference in statistical values, even if they all reflect survival, might be an important factor. It is also important to note that our review is mainly based on case reports, and to a lesser extent case series, being inevitably susceptible to selection and publication bias. Case reports focus on rare cases with a tendency for positive outcomes, in contrast to single-/multi-center studies. Thus, in terms of accuracy, single-/multi-center studies are more reliable. Another major factor is that previous studies include patients examined from 1959 to 1998 [96], 1983–2007 [97], 1979 to 2012 [8], and 1978 to 2007 [98], namely a broad time range. On the other hand, our case reports have been recently published within the last 15 years. Better and more sensitive diagnostic possibilities have emerged throughout these years, allowing the earlier detection of renal disease and metastases. Early-stage disease detection plays an important role in efficient surgical management. Furthermore, adjuvant treatment schemes are more numerous. Efficient treatment is the milestone of good prognosis.

There are limitations to our review, which lie mainly in the numerous missing data in the analyzed case reports and series, as well as in the heterogeneity of the reported data, for example, imaging and histopathological results. Due to missing data, statistical analyses were based on limited patient numbers, and generalization was not considered feasible. Furthermore, case reports and case series are inherently prone to bias as 1. there is lack of control group, 2. they rely on selectively reported/unusual cases, and inherently have small sample sizes 3. there is no systematic data collection. Such limitations make them particularly vulnerable to selection and publication bias. We could not confirm, for instance, whether metastatic burden and multi-metastatic disease, aforementioned risk factors, affect overall survival. A question for future research is also how initial disease stage and/or grade affect survival after thyroid metastases. It is also under consideration whether new therapeutic approaches offer a survival benefit over the traditional surgical approach. As for the metastases’ features, there is a gap concerning the molecular profile and its similarity with the primary focus. Finally, apart from the pathological characteristics there are no other specific diagnostic characteristics to differentiate metastasis from thyroid primaries. However, this study collects world-wide data and represents a pragmatic situation.

## 5. Conclusions

In summary, we reviewed cases and case series of ccRCC metastases to the thyroid, describing the clinical, pathological, ancillary, and follow-up data, in the light of recent therapeutic schemes. We investigated how the parameters of the primary and metastatic disease affect survival in patients with ccRCC.

Our findings suggest that ccRCC metastasis to the thyroid gland often presents as a clinically noticeable neck mass with suspicious imaging features and does not affect thyroid hormone status. A solitary metastatic pattern and a median size of 3.5 cm are commonly observed. The histological and immunohistochemical features of the primary disease are retained. Other common non-thyroid metastases are seen in the urogenital system, lungs, and pancreas. We calculated the OS from initial diagnosis to be 274.6 months, which is significantly higher than reported in the current literature. However, we could not explain how different factors such as novel therapies affect this finding because the results were statistically insignificant. Finally, we discussed our findings in the context of the current literature.

## Figures and Tables

**Table 1:** Baseline characteristics of the study population.

Characteristic	Overall (N = 189)
**Gender**	
Female	88 (46.6%)
Male	97 (51.3%)
Missing	4 (2.1%)
**Age**	
Mean (SD)	65.1 (10.3)
Median [Min, Max]	65.0 [39.0, 88.0]
**Synchronous neoplasia (other)**	
No	65 (34.4%)
Yes	16 (8.5%)
Missing	108 (57.1%)
**Non-thyroid-metastasis (prior)**	
No	64 (33.9%)
Yes	16 (8.5%)
Missing	109 (57.7%)
**Non-thyroid-metastasis (synchronous)**	
No	65 (34.3%)
Yes	15 (7.9%)
Missing	109 (57.8%)
**Follow-up after thyroid metastasis (months)**	
Mean (SD)	35.6 (35.3)
Median [Min, Max]	24.0 [1.50, 156]
Missing	138 (73.0%)
**Larger tumor dimension (cm)**	
Mean (SD)	3.83 (2.03)
Median [Min, Max]	3.50 [0.800, 10.0]
Missing	91 (48.1%)
**Solitary (S)/Multiple (M)**	
M	29 (15.3%)
S	97 (51.3%)
Missing	63 (33.3%)
**Laterality (R: right, L: left, B: both lobes)**	
B	26 (13.8%)
Isthmus	1 (0.5%)
L	38 (20.1%)
L and isthmus	1 (0.5%)
R	47 (24.9%)
Missing	76 (40.2%)
**Stage**	
I	20 (10.6%)
II	5 (2.6%)
III	12 (6.3%)
IV	7 (3.7%)
Missing	145 (76.8%)
**Grade (WHO/ISUP or Fuhrman) on diagnosis**	
1	4 (2.1%)
2	20 (10.6%)
3	14 (7.4%)
4	3 (1.6%)
Missing	148 (78.3%)
**Kidney disease treatment**	
Partial nephrectomy	3 (1.5%)
Total nephrectomy	37 (19.6%)
Radical nephrectomy	30 (16%)
Total nephrectomy and contralateral adrenalectomy	1 (0.5%)
Missing	118 (62.4%)
**Outcome**	
A	26 (13.8%) *
AWD	10 (5.3%)
AWtD	44 (23.3%)
D	26 (13.8%)
DOD	13 (6.9%)
Missing	70 (36.9%)

*: For manuscripts in which a long follow-up outcome was not clearly reported, but a metastatic disease was reported, these patients were considered alive from diagnosis time till the metastasis time, and thus the number of alive patients including these rises to 86 (45.5%).

## Data Availability

Not applicable.

## Supplementary Materials

The following supporting information can be downloaded at https://www.mdpi.com/article/10.3390/cancers18010057/s1, Figure S1: PRISMA flow diagram of our search strategy; Figure S2: Survival plots for study groups along with number at risk *p*-value for log-rank test and hazard ratio (HR); Table S1: Demographic and clinical patient characteristics; Table S2: ccRCC and thyroid metastases characteristics; Table S3: Analytical presentation of the metastases’ imaging features; Table S4: Histopathological features of ccRCC metastases; Table S5: Results for the evaluation of study characteristics in the OS (from initial diagnosis); Table S6: Results for the evaluation of study characteristics in the OS (from diagnosis of metastasis). Reference [106] is cited in the supplementary materials.
