# Supplementary material for: Clear Cell Renal Cell Carcinoma Metastasis to the Thyroid: A Narrative Review of the Literature
Source: Cancers (Basel). 2025 Dec 24;18(1):57. doi: 10.3390/cancers18010057 (PMC12785063; doi:10.3390/cancers18010057)
Supplement: Supplementary file 1 [file cancers-18-00057-s001.zip › Figure S1.docx]

**Identification of studies via databases and registers**

Records identified from*:

Databases (n = 2888)

PubMed n = 510

Scopus n = 1729

Web of Science n = 649

Records removed *before screening*:

Duplicate records removed (n = 896)

**Identification**

Records excluded**

(n = 356)

Records screened

(n = 1992)

**Screening**

Reports not retrieved

(n = 20)

Full-text reports sought for retrieval

(n = 138)

Reports excluded (n = 42):

- Insufficient patient data (n = 10)
- Cumulative patient data (n = 22)
- Reviews and Systematic reviews (n = 10)

Reports assessed for eligibility

(n = 118)

Reports retrieved from snowballing

(n = 1)

**Included**

Studies included in review

(n = 77)

**Figure S1.** PRISMA flow diagram of our search strategy.

Source: Page MJ, et al. BMJ 2021;372:n71. doi: 10.1136/bmj.n71.

This work is licensed under CC BY 4.0. To view a copy of this license, visit <https://creativecommons.org/licenses/by/4.0/>
