# Supplementary material for: Clear Cell Renal Cell Carcinoma Metastasis to the Thyroid: A Narrative Review of the Literature
Source: Cancers (Basel). 2025 Dec 24;18(1):57. doi: 10.3390/cancers18010057 (PMC12785063; doi:10.3390/cancers18010057)
Supplement: Supplementary file 1 [file cancers-18-00057-s001.zip › Figure S2.docx]

|  |  | |
| --- | --- | --- |
|  |  | |
|  | |  |

Figure S2. Survival plots for study groups along with number at risk p-value for log-rank test and hazard ratio (HR).
