# Supplementary material for: Clear Cell Renal Cell Carcinoma Metastasis to the Thyroid: A Narrative Review of the Literature
Source: Cancers (Basel). 2025 Dec 24;18(1):57. doi: 10.3390/cancers18010057 (PMC12785063; doi:10.3390/cancers18010057)
Supplement: Supplementary file 1 [file cancers-18-00057-s001.zip › Table S1.docx]

| Table S1. Demographic and clinical patient characteristics. | | | | | | | | | | | | | |
| --- | --- | --- | --- | --- | --- | --- | --- | --- | --- | --- | --- | --- | --- |
| Author | Year | N | Gender | Age | Symptomatology | Physical examination | Synchronous neoplasia | Hormone status | Metastasis treatment | Follow-up (months) | Recurrence | Interval to first recurrence (months) | Outcome |
| Abbasii  et al. | 2018 | 1 | Male | 68 | Compressive symptomatology  *dysphagia odynophagia dysphonia*  *chest pain* | Painless, palpable thyroid gland | No | N/A | Total thyroidectomy | 1.5 | N/A | N/A | N/A |
| Abdel-Aziz  et al. | 2017 | 1 | Female | 81 | Compressive symptomatology  *dysphagia* | N/A | EA | N/A | N/A | N/A | N/A | N/A | N/A |
| Abdul-Hadi  et al. | 2022 | 1 | Female | 88 | Compressive symptomatology  *dysphagia*  *stridor* | Indurated, palpable neck mass | No | N/A | Chemotherapy (N/A scheme) | N/A | N/A | N/A | N/A |
| Al Abdrabalnabi  et al. | 2019 | 1 | Female | 51 | N/A | N/A | No | N/A | Total thyroidectomy | N/A | Forearm  Pancreas  Kidney | 6 | AWtD |
| Albandar  et al. | 2017 | 1 | Female | 45 | Symptomatology from non-thyroid metastases  *brain* | Findings related to non-thyroid metastases  (brain) | No | N/A | N/A | N/A | Brain | N/A | N/A |
| Alberto  et al. | 2024 | 1 | Male | 66 | Compressive symptomatology  *dysphagia* | Palpable thyroid gland | No | N/A | Total thyroidectomy | N/A | No | No | AWtD |
| Alzahrani  et al. | 2021 | 1 | Female | 46 | Compressive symptomatology  *dysphagia* | Palpable thyroid mass | No | N/A | Total thyroidectomy | 60 | Forearm Pancreas  Kidney | 12 | AWtD |
| Aydogdu  et al. | 2024 | 2 | Female | 64 | N/A | N/A | No | N/A | Total thyroidectomy | 44 | No | No | D |
|  |  |  | Female | 64 | N/A | N/A | No | N/A | Total thyroidectomy | 10 | No | No | D |
| Babar  et al. | 2019 | 1 | Male | 79 | Symptomatology from non-thyroid metastases  *urogenital* | N/A | No | N/A | TKI (sunitinib)  immunotherapy (nivolumab) | 24 | No | No | AWD |
| Badawi  et al. | 2022 | 1 | Female | 63 | N/A | N/A | PTC | N/A | Total thyroidectomy | N/A | N/A | N/A | N/A |
| Balta  et al. | 2022 | 1 | Male | 62 | N/A | N/A | N/A | N/A | Chemotherapy (N/A scheme) | N/A | N/A | N/A | AWD |
| Bayraktar  et al. | 2017 | 1 | Female | 52 | Neck mass | N/A | No | Euthyroid | Total thyroidectomy  TKI (pazopanib)  interferon a | 12 | No | No | AWD |
| Bokhari  et al. | 2017 | 1 | Male | 74 | Asymptomatic | Unremarkable | No | N/A | Lobectomy/Hemithyroidectomy | 26 | No | No | AWtD |
| Bruckschen  et al. | 2021 | 1 | Male | 79 | Asymptomatic | Unremarkable | No | E fT4 | TKI (pazobanib) | 12 | Abdomen | 12 | AWD |
| Cesaretti  et al. | 2013 | 3 | Female | 64 | N/A | Palpable, indurated neck mass | No | N/A | Total thyroidectomy | 60 | Head and Neck area | 24 | DOD |
|  |  |  | Male | 48 | Thyroid enlargement with compressive symptomatology *dyspnea dysphonia* | Palpable, indurated neck mass | No | N/A | Lobectomy/Hemithyroidectomy and laterocervical lymphadenectomy | 21 | N/A | N/A | D |
|  |  |  | Male | 71 | Compressive symptomatology  *dysphagia dyspnea* | Palpable neck mass | No | N/A | Total thyroidectomy | 60 | Kidney | N/A | AWtD |
| Chara  et al. | 2011 | 1 | Male | 49 | Asymptomatic | Unremarkable | No | Euthyroid | Lobectomy/Hemithyroidectomy | 132 | Pancreas Duodenum Lung | 72 | AWtD |
| Chin  et al. | 2011 | 1 | Female | 80 | Neck mass | Palpable thyroid nodule | No | N/A | Lobectomy/Hemithyroidectomy | 60 | No | No | AWtD |
| Cilengir  et al. | 2016 | 1 | Male | 56 | N/A | N/A | No | N/A | N/A | N/A | N/A | N/A | N/A |
| Citgez  et al. | 2011 | 1 | Male | 50 | Neck enlargement | Palpable thyroid gland | No | N/A | Total thyroidectomy | N/A | No | No | AWtD |
| Connolly | 2018 | 1 | Male | 84 | Compressive symptomatology  *dyspnea*  *voice hoarseness* | N/A | No | Euthyroid | Inferior thyroid artery embolization and surgical debulking | N/A | N/A | N/A | AWtD |
| D' Angelo  et al. | 2014 | 1 | Male | 57 | N/A | N/A | N/A | N/A | Total thyroidectomy | N/A | N/A | N/A | N/A |
| Demir  et al. | 2012 | 1 | Male | 64 | Neck enlargement with compressive symptomatology  *dyspnea* | N/A | No | Euthyroid | Radical thyroidectomy  interferon a | 12 | No | No | AWtD |
| Di Furia  et al. | 2017 | 1 | Male | 53 | Asymptomatic | Palpable thyroid nodule | No | Euthyroid | Total thyroidectomy | 24 | No | No | AWtD |
| Di Stasi  et al. | 2013 | 1 | Male | 72 | Compressive symptomatology  *dyspnea*  *voice hoarseness* | Palpable thyroid gland | No | Euthyroid | Lobectomy/Hemithyroidectomy and lymph node dissection | 5 | No | No | D |
| Falcone  et al. | 2018 | 1 | Female | 57 | Asymptomatic | Unremarkable | No | N/A | Total thyroidectomy | N/A | No | No | N/A |
| Fei  et al. | 2023 | 1 | Male | 55 | Asymptomatic | Unremarkable | PTC | N/A | Radical thyroidectomy  TKI (sunitinib) | 14 | No | No | AWtD |
| Foppiani  et al. | 2015 | 1 | Male | 74 | Compressive symptomatology  *neck pain* | Palpable, painless thyroid mass | No | Hyperthytoid  *De TSH*  *E fT3, fT4* | Total thyroidectomy | 24 | No | No | AWtD |
| García-Trujillo  et al. | 2024 | 1 | Female | 68 | Neck mass with compressive symptomatology  *dysphagia* | Palpable thyroid nodule | No | N/A | Total thyroidectomy | 18 | Pancreas Lung | 12 | AWD |
| Gawlik  et al. | 2023 | 1 | Female | 63 | Asymptomatic | Palpable thyroid gland | PTC | N/A | Lobectomy/Hemithyroidectomy | 24 | No | No | AWtD |
| Geisbush  et al. | 2019 | 1 | Male | 66 | Compressive symptomatology  *chest pain* | N/A | No | Euthyroid | Palliative radiatiotherapy | N/A | N/A | N/A | N/A |
| Gheorghiu  et al. | 2016 | 1 | Female | 82 | Neck mass with systematic symptomatology  *weight loss* | Palpable, painless, indurated thyroid gland | No | Euthyroid | Total thyroidectomy  mTOR inhibitor (temsirolimus) | N/A | No | No | AWtD |
| Habibullah  et al. | 2020 | 1 | Female | 79 | Neck enlargement | Palpable, painless, indurated neck mass | No | Euthyroid | Total thyroidectomy | 4 | No | No | AWtD |
| Hellums  et al. | 2023 | 1 | Male | 69 | N/A | Palpable thyroid mass | No | N/A | Lobectomy/Hemithyroidectomy | 14 | No | No | AWtD |
| Hryshchyshyn  et al. | 2024 | 1 | Female | 58 | Neck mass with compressive symptomatology  *dysphagia*  *cough* | N/A | No | Subclinically hypothyroid  *E TSH*  *N fT4* | Total thyroidecomy  TKI (sunitinib) | 72 | Renal bed Adrenal Abdominal wall | 48 | AWtD |
| Jackson  et al. | 2017 | 7 | Male | 61 | N/A | N/A | N/A | N/A | Total thyroidectomy | N/A | N/A | N/A | A |
|  |  |  | Male | 50 | N/A | N/A | N/A | N/A | Kinase inhibitor (sorafenib)  TKI (sunitinib)  mTOR inhibitor (temsirolimus) | N/A | N/A | N/A | D |
|  |  |  | Female | 57 | N/A | N/A | N/A | N/A | Radiation  TKI (sunitinib)  mTOR inhibitor (temsirolimus) | N/A | N/A | N/A | D |
|  |  |  | Male | 58 | N/A | N/A | N/A | N/A | Total thyroidectomy  TKI (sunitinib, pazopanib)  mTOR inhibitor (temsirolimus) | N/A | N/A | N/A | A |
|  |  |  | Male | 53 | N/A | N/A | N/A | N/A | Total thyroidectomy  TKI (pazopanib) | N/A | N/A | N/A | A |
|  |  |  | Male | 49 | N/A | N/A | N/A | N/A | Total thyroidectomy | N/A | N/A | N/A | A |
|  |  |  | Female | 39 | N/A | N/A | N/A | N/A | Total thyroidectomy | N/A | N/A | N/A | A |
| Jha  et al. | 2016 | 1 | Male | 87 | Asymptomatic | None | No | N/A | N/A | N/A | N/A | N/A | N/A |
| Jia  et al. | 2023 | 3 | Female | 78 | Compressive symptomatology  *dyspnea*  *voice hoarseness* | Palpable, indurated thyroid nodule | No | Subclinically hypothyroid  *E TSH*  *N fT3, fT4* | Total thyroidectomy | N/A | N/A | N/A | N/A |
|  |  |  | Male | 54 | Goiter with compressive symptomatology | Palpable thyroid gland | No | Euthyroid | Lobectomy/Hemithyroidectomy | N/A | N/A | N/A | N/A |
|  |  |  | Female | 65 | Goiter with compressive symptomatology | Palpable thyroid gland | No | Euthyroid | Lobectomy/Hemithyroidectomy | N/A | N/A | N/A | N/A |
| Kaliszewski  et al. | 2019 | 9 | Female | 69 | N/A | N/A | N/A | N/A | Total thyroidectomy | N/A | Lung | N/A | DOD |
|  |  |  | Male | 81 | N/A | N/A | N/A | N/A | Total thyroidectomy | N/A | No | N/A | D |
|  |  |  | Female | 72 | N/A | N/A | N/A | N/A | Lobectomy/Hemithyroidectomy | N/A | N/A | N/A | DOD |
|  |  |  | Female | 61 | N/A | N/A | N/A | N/A | Total thyroidectomy | N/A | Kidney | N/A | AWtD |
|  |  |  | Female | 77 | N/A | N/A | N/A | N/A | Total thyroidectomy | N/A | Lung  Brain | N/A | DOD |
|  |  |  | Female | 67 | N/A | N/A | N/A | N/A | Total thyroidectomy | N/A | No | N/A | AWtD |
|  |  |  | Male | 43 | N/A | N/A | N/A | N/A | Tracheostomy/surgical biopsy | N/A | N/A | N/A | DOD |
|  |  |  | Female | 78 | N/A | N/A | N/A | N/A | Total thyroidectomy | N/A | Lung | N/A | DOD |
|  |  |  | Female | 64 | N/A | N/A | N/A | N/A | Total thyroidectomy | N/A | No | N/A | AWtD |
| Kefeli et al. | 2016 | 1 | Female | 80 | Asymptomatic | Palpable thyroid gland | PTC | Euthyroid | Lobectomy/Hemithyroidectomy | N/A | N/A | N/A | N/A |
| Khalafi-Nezhad  et al. | 2024 | 1 | Female | 75 | Neck mass | Palpable thyroid gland | No | Euthyroid | Total thyroidectomy | 24 | No | No | AWtD |
| Khan  et al. | 2018 | 1 | Female | 81 | Compressive and systematic symptomatology  *dysphagia*  *loss of appetite fatigue* | Unremarkable | EA | N/A | Palliative therapy | 2 | No | No | DOD |
| Kobayashi  et al. | 2015 | 7 | Female | 70 | N/A | N/A | N/A | N/A | Lobectomy/Hemithyroidectomy | N/A | N/A | N/A | N/A |
|  |  |  | Male | 77 | N/A | N/A | N/A | N/A | Lobectomy/Hemithyroidectomy | N/A | N/A | N/A | N/A |
|  |  |  | Male | 72 | N/A | N/A | N/A | N/A | Lobectomy/Hemithyroidectomy | N/A | N/A | N/A | N/A |
|  |  |  | Female | 58 | N/A | N/A | N/A | N/A | Lobectomy/Hemithyroidectomy | N/A | N/A | N/A | N/A |
|  |  |  | Female | 67 | N/A | N/A | N/A | N/A | Total thyroidectomy | N/A | N/A | N/A | N/A |
|  |  |  | Female | 63 | N/A | N/A | N/A | N/A | Lobectomy/Hemithyroidectomy | N/A | N/A | N/A | N/A |
|  |  |  | Male | 57 | N/A | N/A | N/A | N/A | Lobectomy/Hemithyroidectomy | N/A | N/A | N/A | N/A |
| Krishnamurthy  et al. | 2014 | 1 | Male | 67 | Neck enlargement with compressive symptomatology  *neck pain*  *dyspnea* | Palpable, indurated thyroid gland | PTmC | N/A | Total thyroidectomy  adjuvant TKI (sunitinib) | 4 | No | No | AWD |
| Lee  et al. | 2011 | 1 | Male | 77 | Asymptomatic | Palpable, painless thyroid mass | No | Euthyroid | Total thyroidectomy | N/A | N/A | N/A | N/A |
| Lieder  et al. | 2017 | 3 | N/A | 58 | N/A | N/A | N/A | N/A | Total thyroidectomy | 24 | N/A | N/A | A |
|  |  |  | N/A | 48 | N/A | N/A | N/A | N/A | Total thyroidectomy chemotherapy (N/A scheme)  TKI (sunitinib) | 27 | N/A | N/A | A |
|  |  |  | N/A | 73 | N/A | N/A | N/A | N/A | Total thyroidectomy  radiotherapy | 86 | N/A | N/A | A |
| Liu  et al. | 2025 | 1 | Female | 60 | Asymptomatic | Unremarkable | HTTT | Euthyroid | Total thyroidectomy  adjuvant TKI (N/A scheme)  immunotherapy (N/A scheme) | 12 | No | No | AWtD |
| Lo  et al. | 2015 | 1 | Male | 60 | Goiter | Palpable thyroid nodule | miHCC | N/A | Lobectomy/Hemithyroidectomy chemotherapy (N/A scheme) | N/A | N/A | N/A | AWD |
| Macedo-Alves  et al. | 2015 | 1 | Female | 80 | Compressive symptomatology  *neck pain*  *voice hoarseness* | Palpable thyroid nodule | pNET | Euthyroid | Lobectomy/Hemithyroidectomy | 11 | No | No | AWtD |
| Medas  et al. | 2013 | 1 | Female | 62 | Goiter | Palpable thyroid gland | FTA | N/A | Total thyroidectomy | N/A | N/A | N/A | AWtD |
| Moghaddam  et al. | 2013 | 1 | Female | 66 | N/A | N/A | No | N/A | Lobectomy/Hemithyroidectomy | N/A | N/A | N/A | N/A |
| Mohammadi  et al. | 2014 | 1 | Male | 58 | Neck mass with compressive and systematic symptomatology *neck pain*  *weight loss* | Palpable, painless, indurated thyroid mass | No | Euthyroid | N/A | N/A | N/A | N/A | N/A |
| Moradi T. et al. | 2020 | 1 | Male | 65 | N/A | N/A | No | N/A | Total thyroidectomy | N/A | N/A | N/A | N/A |
| Nixon  et al. | 2011 | 10 | Female | 56 | Neck mass | N/A | No | N/A | Total thyroidectomy | N/A | N/A | N/A | N/A |
|  |  |  | Female | 58 | Neck mass | N/A | No | N/A | Lobectomy/Hemithyroidectomy | N/A | N/A | N/A | D |
|  |  |  | Male | 60 | Asymptomatic | N/A | No | N/A | Total thyroidectomy | N/A | N/A | N/A | N/A |
|  |  |  | Male | 69 | Neck mass | N/A | No | N/A | Lobectomy/Hemithyroidectomy | N/A | N/A | N/A | D |
|  |  |  | Female | 71 | Neck mass | N/A | No | N/A | Total thyroidectomy | N/A | N/A | N/A | D |
|  |  |  | Female | 73 | Neck mass | N/A | No | N/A | Total thyroidectomy | N/A | N/A | N/A | D |
|  |  |  | Female | 76 | Neck mass | N/A | No | N/A | N/A | N/A | N/A | N/A | D |
|  |  |  | Male | 76 | Neck mass | N/A | No | N/A | Lobectomy/Hemithyroidectomy | N/A | N/A | N/A | D |
|  |  |  | Male | 83 | Neck mass | N/A | No | N/A | Total thyroidectomy | N/A | N/A | N/A | D |
|  |  |  | Female | 83 | Neck mass | N/A | No | N/A | Total thyroidectomy | N/A | N/A | N/A | D |
| Rahman  et al. | 2017 | 1 | Female | 69 | Neck mass | N/A | No | N/A | N/A | N/A | N/A | N/A | N/A |
| Ramírez-Plaza  et al. | 2015 | 1 | Female | 62 | Asymptomatic | Palpable thyroid mass | No | N/A | Radical thyroidectomy | 48 | No | No | AWtD |
| Ricci  et al. | 2021 | 3 | Female | 61 | Asymptomatic | Unremarkable | No | N/A | Radical thyroidectomy | N/A | Lung | 36 | AWtD |
|  |  |  | Male | 76 | Neck mass with compressive symptomatology  *neck pain dysphagia*  *cough* | N/A | No | N/A | Total thyroidectomy | N/A | N/A | N/A | AWtD |
|  |  |  | Female | 78 | Neck mass | Palpable thyroid nodule | No | N/A | Total thyroidectomy | N/A | N/A | N/A | N/A |
| Russel  et al. | 2016 | 10 | Male | 68 | N/A | N/A | N/A | N/A | Total thyroidectomy | 48 | No | N/A | A |
|  |  |  | Male | 45 | N/A | N/A | N/A | N/A | Lobectomy/Hemithyroidectomy | 156 | Yes | N/A | A |
|  |  |  | Female | 66 | N/A | N/A | N/A | N/A | Lobectomy/Hemithyroidectomy | 132 | No | N/A | A |
|  |  |  | Female | 56 | N/A | N/A | N/A | N/A | Lobectomy/Hemithyroidectomy | 14 | No | N/A | A |
|  |  |  | Male | 55 | N/A | N/A | N/A | N/A | Lobectomy/Hemithyroidectomy | 4 | Yes | N/A | A |
|  |  |  | Male | 65 | N/A | N/A | N/A | N/A | Total thyroidectomy | 96 | No | N/A | D |
|  |  |  | Male | 67 | N/A | N/A | N/A | N/A | Total thyroidectomy | 36 | Yes | N/A | A |
|  |  |  | Male | 74 | N/A | N/A | N/A | N/A | Lobectomy/Hemithyroidectomy | 4 | Yes | N/A | A |
|  |  |  | Male | 59 | N/A | N/A | N/A | N/A | Lobectomy/Hemithyroidectomy | 72 | No | N/A | D |
|  |  |  | Male | 65 | N/A | N/A | N/A | N/A | Lobectomy/Hemithyroidectomy | 15 | Yes | N/A | A |
| Sarkar  et al. | 2024 | 1 | Male | 87 | Neck mass | Palpable, indurated thyroid nodule | No | Euthyroid | Lobectomy/Hemithyroidectomy | N/A | N/A | N/A | A |
| Sepherd  et al. | 2022 | 1 | Female | 81 | Thyroid nodule size increase | Palpable, painless thyroid nodule | No | Euthyroid | Lobectomy/Hemithyroidectomy | 4 | No | No | AWtD |
| Shi  et al. | 2015 | 1 | Female | 56 | Preauricular mass | Palpable, painless, indurated neck mass | No | N/A | N/A | 48 | Parotid gland | 24 | AWtD |
| Sindoni  et al. | 2010 | 1 | N/A | 79 | Neck mass | Palpable thyroid gland | No | Euthyroid | Total thyroidectomy | 26 | No | No | AWtD |
| Solmaz  et al. | 2017 | 1 | Female | 64 | Goiter | Palpable thyroid nodules | No | Euthyroid | Total thyroidectomy | N/A | N/A | N/A | N/A |
| Song  et al. | 2017 | 8 | Male | 76 | Neck mass | N/A | N/A | N/A | N/A | N/A | N/A | N/A | N/A |
|  |  |  | Male | 66 | Neck mass | N/A | N/A | N/A | N/A | N/A | N/A | N/A | N/A |
|  |  |  | Female | 59 | Neck mass | N/A | N/A | N/A | N/A | N/A | N/A | N/A | N/A |
|  |  |  | Male | 49 | Neck mass | N/A | N/A | N/A | N/A | N/A | N/A | N/A | N/A |
|  |  |  | Male | 62 | Asymptomatic | N/A | N/A | N/A | N/A | N/A | N/A | N/A | N/A |
|  |  |  | Female | 51 | Asymptomatic | N/A | N/A | N/A | N/A | N/A | N/A | N/A | N/A |
|  |  |  | Male | 49 | Neck mass | N/A | N/A | N/A | N/A | N/A | N/A | N/A | N/A |
|  |  |  | Male | 66 | Neck mass | N/A | N/A | N/A | N/A | N/A | N/A | N/A | N/A |
| Surov  et al. | 2016 | 26 | Male | 70 | Neck mass | N/A | N/A | N/A | N/A | N/A | N/A | N/A | N/A |
|  |  |  | Male | 51 | Neck mass | N/A | N/A | N/A | N/A | N/A | N/A | N/A | N/A |
|  |  |  | Female | 70 | Neck mass with compressive symptomatology  *dyspnea* | N/A | N/A | N/A | N/A | N/A | N/A | N/A | N/A |
|  |  |  | Female | 70 | Asymptomatic | N/A | N/A | N/A | N/A | N/A | N/A | N/A | N/A |
|  |  |  | Female | 64 | Asymptomatic | N/A | N/A | N/A | N/A | N/A | N/A | N/A | N/A |
|  |  |  | Male | 62 | Neck mass | N/A | N/A | N/A | N/A | N/A | N/A | N/A | N/A |
|  |  |  | Female | 64 | Neck mass | N/A | N/A | N/A | N/A | N/A | N/A | N/A | N/A |
|  |  |  | Male | 73 | Neck mass | N/A | N/A | N/A | N/A | N/A | N/A | N/A | N/A |
|  |  |  | Male | 69 | Neck mass | N/A | N/A | N/A | N/A | N/A | N/A | N/A | N/A |
|  |  |  | Female | 82 | Neck mass | N/A | N/A | N/A | N/A | N/A | N/A | N/A | N/A |
|  |  |  | Male | 73 | Neck mass | N/A | N/A | N/A | N/A | N/A | N/A | N/A | N/A |
|  |  |  | Male | 72 | Neck mass | N/A | N/A | N/A | N/A | N/A | N/A | N/A | N/A |
|  |  |  | Female | 72 | Neck mass with compressive symptomatology  *dysphagia* | N/A | N/A | N/A | N/A | N/A | N/A | N/A | N/A |
|  |  |  | Female | 72 | Neck mass | N/A | N/A | N/A | N/A | N/A | N/A | N/A | N/A |
|  |  |  | Male | 61 | Neck mass | N/A | N/A | N/A | N/A | N/A | N/A | N/A | N/A |
|  |  |  | Female | 60 | Neck mass | N/A | N/A | N/A | N/A | N/A | N/A | N/A | N/A |
|  |  |  | Female | 68 | Neck mass | N/A | N/A | N/A | N/A | N/A | N/A | N/A | N/A |
|  |  |  | Female | 77 | Neck mass | N/A | N/A | N/A | N/A | N/A | N/A | N/A | N/A |
|  |  |  | Male | 66 | Neck mass | N/A | N/A | N/A | N/A | N/A | N/A | N/A | N/A |
|  |  |  | Male | 63 | Neck mass | N/A | N/A | N/A | N/A | N/A | N/A | N/A | N/A |
|  |  |  | Male | 66 | Neck mass | N/A | N/A | N/A | N/A | N/A | N/A | N/A | N/A |
|  |  |  | Male | 65 | Neck mass | N/A | N/A | N/A | N/A | N/A | N/A | N/A | N/A |
|  |  |  | Female | 54 | Neck mass with compressive symptomatology  *dysphagia* | N/A | N/A | N/A | N/A | N/A | N/A | N/A | N/A |
|  |  |  | Male | 69 | Neck mass | N/A | N/A | N/A | N/A | N/A | N/A | N/A | N/A |
|  |  |  | Female | 73 | Neck mass | N/A | N/A | N/A | N/A | N/A | N/A | N/A | N/A |
|  |  |  | Female | 69 | Neck mass | N/A | N/A | N/A | N/A | N/A | N/A | N/A | N/A |
| Tadisina  et al. | 2024 | 1 | Male | 65 | Asymptomatic | Palpable thyroid gland | PTC | Euthyroid | Total thyroidectomy | N/A | No | No | AWtD |
| Tang  et al. | 2022 | 2 | Female | 69 | Asymptomatic | N/A | N/A | N/A | Total thyroidectomy | N/A | N/A | N/A | N/A |
|  |  |  | Male | 66 | Neck enlargement | N/A | N/A | N/A | N/A | N/A | N/A | N/A | N/A |
| Tian  et al. | 2020 | 1 | Male | 57 | Compressive symptomatology  *discomfort* | Palpable thyroid gland | FTA | Euthyroid | Radical thyroidectomy | 24 | No | No | AWtD |
| Tjahjono  et al. | 2021 | 15 | Male | 64 | N/A | N/A | N/A | N/A | Lobectomy/Hemithyroidectomy | N/A | N/A | N/A | D |
|  |  |  | Female | 52 | N/A | N/A | N/A | N/A | Lobectomy/Hemithyroidectomy | N/A | N/A | N/A | A |
|  |  |  | Male | 60 | N/A | N/A | N/A | N/A | Lobectomy/Hemithyroidectomy | N/A | N/A | N/A | A |
|  |  |  | Female | 63 | N/A | N/A | N/A | N/A | Lobectomy/Hemithyroidectomy | N/A | N/A | N/A | A |
|  |  |  | Female | 53 | N/A | N/A | N/A | N/A | Total thyroidectomy | N/A | N/A | N/A | A |
|  |  |  | Male | 46 | N/A | N/A | N/A | N/A | Total thyroidectomy | N/A | N/A | N/A | A |
|  |  |  | Male | 51 | N/A | N/A | N/A | N/A | N/A | N/A | N/A | N/A | D |
|  |  |  | Male | 50 | N/A | N/A | N/A | N/A | Lobectomy/Hemithyroidectomy | N/A | N/A | N/A | A |
|  |  |  | Male | 56 | N/A | N/A | N/A | N/A | Lobectomy/Hemithyroidectomy | N/A | N/A | N/A | D |
|  |  |  | Female | 66 | N/A | N/A | N/A | N/A | N/A | N/A | N/A | N/A | D |
|  |  |  | Male | 50 | N/A | N/A | N/A | N/A | N/A | N/A | N/A | N/A | A |
|  |  |  | Female | 61 | N/A | N/A | N/A | N/A | Lobectomy/Hemithyroidectomy | N/A | N/A | N/A | D |
|  |  |  | Female | 65 | N/A | N/A | N/A | N/A | Total thyroidectomy | N/A | N/A | N/A | A |
|  |  |  | Female | 57 | N/A | N/A | N/A | N/A | Total thyroidectomy | N/A | N/A | N/A | A |
|  |  |  | Male | 62 | N/A | N/A | N/A | N/A | Lobectomy/Hemithyroidectomy | N/A | N/A | N/A | D |
| Valdez  et al. | 2014 | 1 | Female | 78 | Asymptomatic | Palpable thyroid nodule | No | Euthyroid | Total thyroidectomy | N/A | N/A | N/A | N/A |
| Vandermegel | 2021 | 1 | Male | 60 | Neck mass | Palpable, indurated thyroid nodule | No | Euthyroid | Total thyroidectomy  radiotherapy | 6 | No | No | AWtD |
| Velez Torres  et al. | 2022 | 17 | Female | 83 | Neck mass | N/A | N/A | N/A | Embolization | N/A | N/A | N/A | N/A |
|  |  |  | Male | 64 | Neck mass | N/A | N/A | N/A | Total thyroidectomy | N/A | N/A | N/A | DOD |
|  |  |  | Male | 53 | Neck mass | N/A | N/A | N/A | Lobectomy/Hemithyroidectomy | N/A | N/A | N/A | AWtD |
|  |  |  | Female | 69 | Neck mass | N/A | N/A | N/A | Total thyroidectomy | N/A | N/A | N/A | N/A |
|  |  |  | Male | 74 | Neck mass | N/A | N/A | N/A | Total thyroidectomy | N/A | N/A | N/A | D |
|  |  |  | Female | 59 | Neck mass | N/A | N/A | N/A | Total thyroidectomy | N/A | N/A | N/A | AWtD |
|  |  |  | Female | 45 | N/A | N/A | N/A | N/A | Chemotherapy (N/A scheme) | N/A | N/A | N/A | DOD |
|  |  |  | Male | 75 | N/A | N/A | N/A | N/A | Radiotherapy | N/A | N/A | N/A | AWD |
|  |  |  | Male | 65 | N/A | N/A | N/A | N/A | Chemotherapy (N/A scheme) | N/A | N/A | N/A | DOD |
|  |  |  | Female | 66 | Thyroid enlargement | N/A | N/A | N/A | Lobectomy/Hemithyroidectomy | N/A | N/A | N/A | AWtD |
|  |  |  | Male | 63 | Thyroid lobe enlargement | N/A | N/A | N/A | Total thyroidectomy | N/A | N/A | N/A | AWtD |
|  |  |  | Male | 68 | Thyroid nodules size increase | N/A | N/A | N/A | Total thyroidectomy | N/A | N/A | N/A | AWtD |
|  |  |  | Male | 88 | Compressive symptomatology  *dyspnea* | N/A | N/A | N/A | Palliative therapy | N/A | N/A | N/A | AWD |
|  |  |  | Male | 82 | Neck mass | N/A | N/A | N/A | Total thyroidectomy | N/A | N/A | N/A | DOD |
|  |  |  | Male | 75 | N/A | N/A | N/A | N/A | Lobectomy/Hemithyroidectomy immunotherapy (N/A scheme) | N/A | N/A | N/A | AWD |
|  |  |  | Male | 81 | Asymptomatic | N/A | N/A | N/A | Lobectomy/Hemithyroidectomy | N/A | N/A | N/A | DOD |
|  |  |  | Male | 58 | Neck mass | N/A | N/A | N/A | Lobectomy/Hemithyroidectomy | N/A | N/A | N/A | AWtD |
| Wong  et al. | 2017 | 1 | Female | 58 | Neck enlargement | N/A | Metastatic to the thyroid breast carcinoma | N/A | Radical thyroidectomy | N/A | N/A | N/A | N/A |
| Xie  et al. | 2023 | 1 | Female | 66 | Thyroid nodules size increase | Palpable thyroid nodule | PTC | Euthyroid | Left thyroidectomy, right partial thyroidectomy and left lateral lymph node dissection | N/A | No | No | AWtD |
| Xie  et al. | 2023 | 2 | Female | 58 | N/A | N/A | N/A | N/A | Total thyroidectomy | 27 | N/A | N/A | D |
|  |  |  | Male | 54 | N/A | N/A | N/A | N/A | Total thyroidectomy | 34 | N/A | N/A | D |
| Xu  et al. | 2024 | 2 | Male | 59 | Asymptomatic | Palpable thyroid gland | No | Subclinically hypothyroid  *E TSH*  *N fT3, fT4* | Radical thyroidectomy | N/A | No | No | AWtD |
|  |  |  | Female | 62 | Neck mass | Palpable thyroid gland | No | Euthyroid | Lobectomy/Hemithyroidectomy | N/A | No | No | AWtD |
| Yamauchi  et al. | 2018 | 1 | Male | 59 | Asymptomatic | Palpable, painless, indurated thyroid mass | No | Euthyroid | Lobectomy/Hemithyroidectomy TKI (sunitinib) | 26 | Lung | 1 | AWtD |
| Zamarrón  et al. | 2013 | 1 | Female | 51 | N/A | N/A | FTmC | N/A | Total thyroidectomy | 72 | Lung Supraclavicular lymph nodes Rhomboid muscle | 12 | DOD |

N/A: non-applicable, EA: Esophageal adenocarcinoma, PTC: Papillary thyroid carcinoma, PTmC: Papillary thyroid microcarcinoma, HTTT: Hyalinizing trabecular thyroid tumor, miHCC: minimally invasive Hurthle cell carcinoma, pNET: pancreatic neuroendocrine tumor, FTA: Follicular thyroid adenoma, FTmC: Follicular thyroid microcarcinoma, E: elevated, De: decreased, N: normal, f: free, A: alive, D: deceased, AWD: alive with disease, AWtD: alive without disease, DOD: deceased of disease
