# Supplementary material for: Clear Cell Renal Cell Carcinoma Metastasis to the Thyroid: A Narrative Review of the Literature
Source: Cancers (Basel). 2025 Dec 24;18(1):57. doi: 10.3390/cancers18010057 (PMC12785063; doi:10.3390/cancers18010057)
Supplement: Supplementary file 1 [file cancers-18-00057-s001.zip › Table S2.docx]

| Table S2. ccRCC and thyroid metastases characteristics. | | | | | | | | | | | | |
| --- | --- | --- | --- | --- | --- | --- | --- | --- | --- | --- | --- | --- |
| Study characteristics | | | **ccRCC characteristics** | | | | | | **Thyroid metastases characteristics** | | | |
| Author | **Year** | **N** | **Stage*** | **Grade~** | **Laterality** | **Nephrectomy type** | **Synchronous-to-thyroid metastases** | **Prior-to-thyroid metastases** | **Largest dimension**  **(cm)** | **Distribution** | **Laterality** | **Interval to thyroid metastases**  **(months)** |
| Abbasii  et al. | 2018 | 1 | I  (T1bN0) | 2 | R | Total | Lung  Kidney Mesenteric axis | No | N/A | S | B | 96 |
| Abdel-Aziz  et al. | 2017 | 1 | N/A | N/A | R | Total | No | No | 2.69 | S | R | 132 |
| Abdul-Hadi  et al. | 2022 | 1 | N/A | N/A | R | Total | Lung | No | 5.1 | S | L | 300 |
| Al Abdrabalnabi  et al. | 2019 | 1 | N/A | N/A | R | Radical | Pancreas  Forearm  Kidney | Pancreas | 5.2 | S | L | 120 |
| Albandar  et al. | 2017 | 1 | I  (T1bN0M0) | 3 | R | Partial | Brain | No | N/A | S | L and isthmus | 18 |
| Alberto  et al. | 2024 | 1 | III  (T3aNxM0) | N/A | N/A | Total | No | Lung Mediastinum | 2.3 | M (2) | R | 120 |
| Alzahrani  et al. | 2021 | 1 | I | N/A | R | Radical | Pancreas  Kidney  Forearm | Pancreas | N/A | M (4) | B | 96 |
| Aydogdu  et al. | 2024 | 2 | N/A | N/A | N/A | N/A | No | No | 4 | N/A | N/A | 94 |
|  |  |  | N/A | N/A | N/A | N/A | No | No | 1.7 | N/A | N/A | 43 |
| Babar  et al. | 2019 | 1 | N/A | N/A | L | Radical | Urinary bladder  Kidney Acetabulum  1st rib Retroperitoneum Lung | No | 1.7 | S | R | 336 |
| Badawi  et al. | 2022 | 1 | N/A | N/A | L | Radical | No | Kidney | 1.6 | S | R | 168 |
| Balta  et al. | 2022 | 1 | N/A | N/A | N/A | N/A | N/A | N/A | N/A | N/A | N/A | N/A |
| Bayraktar  et al. | 2017 | 1 | I  (T1bN0M0) | 3 | R | Radical | Lung  Mediastinal lymph nodes | No | 2.4 | S | Isthmus | 108 |
| Bokhari  et al. | 2017 | 1 | IV (T3bNxM1) | 3 | L | Radical | No | Pancreas Adrenal gland  Lung | 1 | S | L | 48 |
| Bruckschen  et al. | 2021 | 1 | N/A | N/A | R | Total | No | No | N/A | N/A | N/A | 204 |
| Cesaretti  et al. | 2013 | 3 | N/A | N/A | R | Total | No | No | 5.5 | S | L | 48 |
|  |  |  | N/A | N/A | L | Total | No | No | N/A | S | R | 96 |
|  |  |  | N/A | N/A | R | Total | No | No | N/A | N/A | N/A | 132 |
| Chara  et al. | 2011 | 1 | II (T2aN0M0) | N/A | L | Radical | No | No | 4 | S | L | 8 |
| Chin  et al. | 2011 | 1 | I  (T1bN0) | 4 | N/A | Radical | No | No | 4 | S | N/A | 96 |
| Cilengir  et al. | 2016 | 1 | IV (T2aNxM1) | 4 | L | Total and controlateral adrenalectomy | Upper abdominal lymph nodes | Adrenal gland | 3 | S | R | N/A |
| Citgez  et al. | 2011 | 1 | I  (T1a) | N/A | R | Total | No | No | 3.7 | M | B | 36 |
| Connolly | 2018 | 1 | N/A | N/A | R | Total | No | No | 9.5 | S | L | 312 |
| D' Angelo  et al. | 2014 | 1 | N/A | N/A | L | Total | N/A | N/A | N/A | S | L | 132 |
| Demir  et al. | 2012 | 1 | N/A | N/A | R | Total | No | No | 6 | M (2) | B | 204 |
| Di Furia  et al. | 2017 | 1 | I  (T1cN0Mx) | 3 | L | Total | No | No | 1.5 | S | R | 12 |
| Di Stasi  et al. | 2013 | 1 | II  (T2a) | N/A | R | Radical | No | No | N/A | S | R | 288 |
| Falcone  et al. | 2018 | 1 | I  (T1b) | N/A | R | Radical | No | No | 1.8 | S | N/A | 108 |
| Fei  et al. | 2023 | 1 | I  (T1aN0M0) | N/A | L | Radical | No | Para-aortic lymph nodes | 1.3 | S | L | 48 |
| Foppiani  et al. | 2015 | 1 | II  (T2N0) | 2 | R | Total | No | No | 6 | S | L | 48 |
| García-Trujillo  et al. | 2024 | 1 | N/A | N/A | N/A | Radical | No | No | 2.5 | S | R | 84 |
| Gawlik  et al. | 2023 | 1 | I  (T1b) | N/A | R | Partial | No | No | N/A | M | L | 12 |
| Geisbush  et al. | 2019 | 1 | N/A | N/A | L | Total | No | No | 3.3 | S | L | 132 |
| Gheorghiu  et al. | 2016 | 1 | N/A | N/A | R | Total | No | No | 10 | S | R | 192 |
| Habibullah  et al. | 2020 | 1 | N/A | N/A | R | Total | No | No | 3 | M | B | 240 |
| Hellums  et al. | 2023 | 1 | N/A | N/A | R | Radical | No | Kidney | 4.7 | S | R | 123 |
| Hryshchyshyn  et al. | 2024 | 1 | I  (T1N0M0) | N/A | N/A | Partial | N/A | N/A | 3.5 | M (2) | B | 156 |
| Jackson  et al. | 2017 | 7 | N/A | N/A | N/A | N/A | N/A | N/A | N/A | N/A | N/A | 45 |
|  |  |  | N/A | N/A | N/A | N/A | N/A | N/A | N/A | N/A | N/A | 17 |
|  |  |  | N/A | N/A | N/A | N/A | N/A | N/A | N/A | N/A | N/A | 2 |
|  |  |  | N/A | N/A | N/A | N/A | N/A | N/A | N/A | N/A | N/A | 99 |
|  |  |  | N/A | N/A | N/A | N/A | N/A | N/A | N/A | N/A | N/A | 17 |
|  |  |  | N/A | N/A | N/A | N/A | N/A | N/A | N/A | N/A | N/A | 54 |
|  |  |  | N/A | N/A | N/A | N/A | N/A | N/A | N/A | N/A | N/A | 173 |
| Jha  et al. | 2016 | 1 | N/A | N/A | L | Total | Lung | No | N/A | M (N/A) | B | 120 |
| Jia  et al. | 2023 | 3 | N/A | N/A | L | Total | No | Pancreas | 4.2 | M (2) | B | 144 |
|  |  |  | N/A | N/A | L | Total | Pancreas | No | 4.5 | S | L | 96 |
|  |  |  | N/A | N/A | L | Total | No | No | 4.5 | S | L | 84 |
| Kaliszewski et al. | 2019 | 9 | N/A | N/A | N/A | N/A | N/A | N/A | N/A | N/A | N/A | N/A |
|  |  |  | N/A | N/A | N/A | N/A | N/A | N/A | N/A | N/A | N/A | N/A |
|  |  |  | N/A | N/A | N/A | N/A | N/A | N/A | N/A | N/A | N/A | N/A |
|  |  |  | N/A | N/A | N/A | N/A | N/A | N/A | N/A | N/A | N/A | N/A |
|  |  |  | N/A | N/A | N/A | N/A | N/A | N/A | N/A | N/A | N/A | N/A |
|  |  |  | N/A | N/A | N/A | N/A | N/A | N/A | N/A | N/A | N/A | N/A |
|  |  |  | N/A | N/A | N/A | N/A | N/A | N/A | N/A | N/A | N/A | N/A |
|  |  |  | N/A | N/A | N/A | N/A | N/A | N/A | N/A | N/A | N/A | N/A |
|  |  |  | N/A | N/A | N/A | N/A | N/A | N/A | N/A | N/A | N/A | N/A |
| Kefeli et al. | 2016 | 1 | N/A | N/A | N/A | Radical | No | No | 4 | S | L | 216 |
| Khalafi-Nezhad  et al. | 2024 | 1 | I  (T1aN0M0) | N/A | R | Radical | No | No | 2.3 | S | L | 156 |
| Khan  et al. | 2018 | 1 | IV (T3bN0M1) | N/A | R | Radical | Kidney | No | 2.8 | S | R | 132 |
| Kobayashi  et al. | 2015 | 7 | N/A | N/A | N/A | N/A | N/A | N/A | 3.6 | S | R | N/A |
|  |  |  | N/A | N/A | N/A | N/A | N/A | N/A | 5.6 | S | R | N/A |
|  |  |  | N/A | N/A | N/A | N/A | N/A | N/A | 3.1 | S | L | N/A |
|  |  |  | N/A | N/A | N/A | N/A | N/A | N/A | 3.9 | S | R | N/A |
|  |  |  | N/A | N/A | N/A | N/A | N/A | N/A | 8.1 | M (2) | B | N/A |
|  |  |  | N/A | N/A | N/A | N/A | N/A | N/A | 4.8 | S | R | N/A |
|  |  |  | N/A | N/A | N/A | N/A | N/A | N/A | 4.8 | S | R | N/A |
| Krishnamurthy  et al. | 2014 | 1 | III  (T3N0M0) | 3 | L | Radical | Lung  Mediastinum Adrenal gland Pancreas | No | 8 | S | R | 180 |
| Lee  et al. | 2011 | 1 | I | N/A | R | Total | Local recurrence | No | 7.2 | M (2) | B | 168 |
| Lieder  et al. | 2017 | 3 | IV (T3aN0M1) | 2 | N/A | Total | N/A | N/A | N/A | N/A | N/A | 5 |
|  |  |  | I  (T1bN0M0) | 2 | N/A | Radical | N/A | N/A | N/A | N/A | N/A | 87 |
|  |  |  | IV (T1aN0M1) | 2 | N/A | Total | N/A | N/A | N/A | N/A | N/A | 40 |
| Liu  et al. | 2025 | 1 | N/A | 1 | L | Radical | No | No | 5.5 | S | L | 24 |
| Lo  et al. | 2015 | 1 | N/A | N/A | N/A | N/A | Lung  Mediastinal lymph nodes Adrenal gland | No | N/A | S | L | 0 |
| Macedo-Alves  et al. | 2015 | 1 | I  (T1N0M0) | 2 | R | Radical | No | No | 2.7 | S | R | 108 |
| Medas  et al. | 2013 | 1 | N/A | N/A | L | Total | No | No | 5.3 | S | L | 72 |
| Moghaddam  et al. | 2013 | 1 | N/A | N/A | N/A | N/A | No | No | 5 | S | R | 72 |
| Mohammadi  et al. | 2014 | 1 | III (T1bN2M0) | N/A | L | Total | No | No | 9 | S | L | 18 |
| Moradi T.  et al. | 2020 | 1 | III  (T3a) | N/A | N/A | Total | No | No | 2.5 | S | R | 36 |
| Nixon  et al. | 2011 | 10 | N/A | N/A | N/A | N/A | No | Yes | 1 | M | N/A | N/A |
|  |  |  | N/A | N/A | N/A | N/A | No | Yes | 1.4 | S | N/A | N/A |
|  |  |  | N/A | N/A | N/A | N/A | No | No | 2.5 | S | N/A | N/A |
|  |  |  | N/A | N/A | N/A | N/A | No | No | 4 | M | N/A | N/A |
|  |  |  | N/A | N/A | N/A | N/A | No | Yes | N/A | S | N/A | N/A |
|  |  |  | N/A | N/A | N/A | N/A | No | No | 1 | M | N/A | N/A |
|  |  |  | N/A | N/A | N/A | N/A | No | Yes | N/A | S | N/A | N/A |
|  |  |  | N/A | N/A | N/A | N/A | No | Yes | N/A | M | N/A | N/A |
|  |  |  | N/A | N/A | N/A | N/A | No | No | 1.3 | M | N/A | N/A |
|  |  |  | N/A | N/A | N/A | N/A | No | Yes | N/A | M | N/A | N/A |
| Rahman  et al. | 2017 | 1 | IV (T1bN0M1) | 2 | L | Radical | No | No | 2 | S | R | 0 |
| Ramírez-Plaza  et al. | 2015 | 1 | IV  (T4N0M1) | 1 | R | Radical | No | No | 2.2 | S | R | 0 |
| Ricci  et al. | 2021 | 3 | III  (T3a) | 3 | L | Radical | No | No | 2.2 | S | R | 120 |
|  |  |  | III (T3aN0M0) | 2 | R | Radical | No | No | 4.5 | S | R | 36 |
|  |  |  | II (T2bN0M0) | 2 | L | Radical | No | No | 3 | S | R | 120 |
| Russel  et al. | 2016 | 10 | N/A | N/A | N/A | N/A | N/A | N/A | N/A | N/A | B | N/A |
|  |  |  | N/A | N/A | N/A | N/A | N/A | N/A | N/A | N/A | R | N/A |
|  |  |  | N/A | N/A | N/A | N/A | N/A | N/A | N/A | N/A | L | N/A |
|  |  |  | N/A | N/A | N/A | N/A | N/A | N/A | N/A | N/A | L | N/A |
|  |  |  | N/A | N/A | N/A | N/A | N/A | N/A | N/A | N/A | R | N/A |
|  |  |  | N/A | N/A | N/A | N/A | N/A | N/A | N/A | N/A | R | N/A |
|  |  |  | N/A | N/A | N/A | N/A | N/A | N/A | N/A | N/A | B | N/A |
|  |  |  | N/A | N/A | N/A | N/A | N/A | N/A | N/A | N/A | B | N/A |
|  |  |  | N/A | N/A | N/A | N/A | N/A | N/A | N/A | N/A | R | N/A |
|  |  |  | N/A | N/A | N/A | N/A | N/A | N/A | N/A | N/A | N/A | N/A |
| Sarkar  et al. | 2024 | 1 | III  (T3b) | 3 | L | Radical | No | No | 5 | S | R | 144 |
| Sepherd  et al. | 2022 | 1 | II  (T2a) | 4 | L | Total | No | No | 5.7 | S | R | 252 |
| Shi  et al. | 2015 | 1 | III | N/A | R | Radical | No | No | N/A | N/A | N/A | 60 |
| Sindoni  et al. | 2010 | 1 | I | N/A | L | Total | No | No | 4 | M (3) | B | 216 |
| Solmaz  et al. | 2017 | 1 | III  (T3) | N/A | L | Total | No | No | 0.8 | M (2) | L | 17 |
| Song  et al. | 2017 | 8 | N/A | N/A | N/A | N/A | N/A | N/A | 5 | S | N/A | 192 |
|  |  |  | N/A | N/A | N/A | N/A | N/A | N/A | 3.5 | M (2) | N/A | 168 |
|  |  |  | N/A | N/A | N/A | N/A | N/A | N/A | 5.1 | S | N/A | 144 |
|  |  |  | N/A | N/A | N/A | N/A | N/A | N/A | 4.1 | S | N/A | 72 |
|  |  |  | N/A | N/A | N/A | N/A | N/A | N/A | 1.5 | S | N/A | 54 |
|  |  |  | N/A | N/A | N/A | N/A | N/A | N/A | 3.2 | S | N/A | 54 |
|  |  |  | N/A | N/A | N/A | N/A | N/A | N/A | 4.9 | S | N/A | 42 |
|  |  |  | N/A | N/A | N/A | N/A | N/A | N/A | 7.4 | S | N/A | 192 |
| Surov  et al. | 2016 | 26 | N/A | N/A | N/A | N/A | N/A | N/A | N/A | N/A | L | 72 |
|  |  |  | N/A | N/A | N/A | N/A | N/A | N/A | N/A | N/A | R | 180 |
|  |  |  | N/A | N/A | N/A | N/A | N/A | N/A | N/A | N/A | R | 24 |
|  |  |  | N/A | N/A | N/A | N/A | N/A | N/A | N/A | N/A | R | 86 |
|  |  |  | N/A | N/A | N/A | N/A | N/A | N/A | N/A | N/A | R | 108 |
|  |  |  | N/A | N/A | N/A | N/A | N/A | N/A | N/A | N/A | L | 156 |
|  |  |  | N/A | N/A | N/A | N/A | N/A | N/A | N/A | N/A | B | 108 |
|  |  |  | N/A | N/A | N/A | N/A | N/A | N/A | N/A | N/A | L | 84 |
|  |  |  | N/A | N/A | N/A | N/A | N/A | N/A | N/A | N/A | B | 204 |
|  |  |  | N/A | N/A | N/A | N/A | N/A | N/A | N/A | N/A | L | 132 |
|  |  |  | N/A | N/A | N/A | N/A | N/A | N/A | N/A | N/A | B | 60 |
|  |  |  | N/A | N/A | N/A | N/A | N/A | N/A | N/A | N/A | L | 168 |
|  |  |  | N/A | N/A | N/A | N/A | N/A | N/A | N/A | N/A | R | 132 |
|  |  |  | N/A | N/A | N/A | N/A | N/A | N/A | N/A | N/A | R | 180 |
|  |  |  | N/A | N/A | N/A | N/A | N/A | N/A | N/A | N/A | B | 0 |
|  |  |  | N/A | N/A | N/A | N/A | N/A | N/A | N/A | N/A | B | 96 |
|  |  |  | N/A | N/A | N/A | N/A | N/A | N/A | N/A | N/A | L | 216 |
|  |  |  | N/A | N/A | N/A | N/A | N/A | N/A | N/A | N/A | L | 48 |
|  |  |  | N/A | N/A | N/A | N/A | N/A | N/A | N/A | N/A | B | 132 |
|  |  |  | N/A | N/A | N/A | N/A | N/A | N/A | N/A | N/A | R | 36 |
|  |  |  | N/A | N/A | N/A | N/A | N/A | N/A | N/A | N/A | L | 132 |
|  |  |  | N/A | N/A | N/A | N/A | N/A | N/A | N/A | N/A | L | 108 |
|  |  |  | N/A | N/A | N/A | N/A | N/A | N/A | N/A | N/A | R | 96 |
|  |  |  | N/A | N/A | N/A | N/A | N/A | N/A | N/A | N/A | R | 144 |
|  |  |  | N/A | N/A | N/A | N/A | N/A | N/A | N/A | N/A | L | 168 |
|  |  |  | N/A | N/A | N/A | N/A | N/A | N/A | N/A | N/A | R | 156 |
| Tadisina  et al. | 2024 | 1 | III  (T3aNx) | N/A | L | Radical | No | No | 3 | S | R | 84 |
| Tang  et al. | 2022 | 2 | N/A | N/A | N/A | N/A | N/A | N/A | 5.8 | S | L | 84 |
|  |  |  | N/A | N/A | N/A | N/A | N/A | N/A | 6.2 | S | L | 120 |
| Tian  et al. | 2020 | 1 | III | 2 | R | Radical | No | No | 4 | S | R | 132 |
| Tjahjono  et al. | 2021 | 15 | N/A | 2 | N/A | N/A | N/A | N/A | N/A | S | N/A | 62 |
|  |  |  | N/A | 1 | N/A | N/A | N/A | N/A | N/A | S | N/A | 120 |
|  |  |  | N/A | 2 | N/A | N/A | N/A | N/A | N/A | S | N/A | 119 |
|  |  |  | N/A | 3 | N/A | N/A | N/A | N/A | N/A | S | N/A | 40 |
|  |  |  | N/A | 2 | N/A | N/A | N/A | N/A | N/A | M | B | 230 |
|  |  |  | N/A | 3 | N/A | N/A | N/A | N/A | N/A | M | B | 220 |
|  |  |  | N/A | 3 | N/A | N/A | N/A | N/A | N/A | S | N/A | 156 |
|  |  |  | N/A | 3 | N/A | N/A | N/A | N/A | N/A | S | N/A | 210 |
|  |  |  | N/A | 2 | N/A | N/A | N/A | N/A | N/A | S | N/A | 120 |
|  |  |  | N/A | 3 | N/A | N/A | N/A | N/A | N/A | M | B | 0 |
|  |  |  | N/A | 3 | N/A | N/A | N/A | N/A | N/A | S | N/A | 0 |
|  |  |  | N/A | 2 | N/A | N/A | N/A | N/A | N/A | S | N/A | 24 |
|  |  |  | N/A | 3 | N/A | N/A | N/A | N/A | N/A | S | N/A | 50 |
|  |  |  | N/A | 2 | N/A | N/A | N/A | N/A | N/A | S | N/A | 84 |
|  |  |  | N/A | 2 | N/A | N/A | N/A | N/A | N/A | S | N/A | 92 |
| Valdez  et al. | 2014 | 1 | I  (T1b) | 1 | R | Total | No | No | 4.4 | S | L | 276 |
| Vandermegel | 2021 | 1 | I  (T1b) | N/A | R | Total | No | No | 4.2 | S | R | 276 |
| Velez Torres  et al. | 2022 | 17 | N/A | N/A | N/A | N/A | N/A | N/A | N/A | S | N/A | 180 |
|  |  |  | N/A | N/A | N/A | N/A | N/A | N/A | 1.6 | S | N/A | 36 |
|  |  |  | N/A | N/A | N/A | N/A | N/A | N/A | 3 | S | N/A | 24 |
|  |  |  | N/A | N/A | N/A | N/A | N/A | N/A | 3.5 | S | N/A | 156 |
|  |  |  | N/A | N/A | N/A | N/A | N/A | N/A | 4.5 | M | N/A | 132 |
|  |  |  | N/A | N/A | N/A | N/A | N/A | N/A | 2 | M | N/A | 60 |
|  |  |  | N/A | N/A | N/A | N/A | N/A | N/A | N/A | S | N/A | 36 |
|  |  |  | N/A | N/A | N/A | N/A | N/A | N/A | 7.6 | S | N/A | 96 |
|  |  |  | N/A | N/A | N/A | N/A | N/A | N/A | 1.2 | S | N/A | 24 |
|  |  |  | N/A | N/A | N/A | N/A | N/A | N/A | 6 | S | N/A | 0 |
|  |  |  | N/A | N/A | N/A | N/A | N/A | N/A | 3 | S | N/A | 240 |
|  |  |  | N/A | N/A | N/A | N/A | N/A | N/A | 3 | M | N/A | 33 |
|  |  |  | N/A | N/A | N/A | N/A | N/A | N/A | 3.5 | S | N/A | 96 |
|  |  |  | N/A | N/A | N/A | N/A | N/A | N/A | 1.7 | S | N/A | 87 |
|  |  |  | N/A | N/A | N/A | N/A | N/A | N/A | 3.5 | S | N/A | 14 |
|  |  |  | N/A | N/A | N/A | N/A | N/A | N/A | 8 | S | N/A | 128 |
|  |  |  | N/A | N/A | N/A | N/A | N/A | N/A | 2.1 | S | N/A | 30 |
| Wong  et al. | 2017 | 1 | N/A | N/A | N/A | Total | No | No | 5.5 | M | B | 96 |
| Xie  et al. | 2023 | 1 | I  (T1b) | 2 | L | Total | Connective tissue adjacent to the thyroid | No | 3.5 | S | L | 96 |
| Xie  et al. | 2023 | 2 | N/A | N/A | N/A | N/A | N/A | N/A | N/A | N/A | B | 180 |
|  |  |  | N/A | N/A | N/A | N/A | N/A | N/A | N/A | N/A | R | 0 |
| Xu  et al. | 2024 | 2 | N/A | N/A | R | Total | No | Retroperitoneum | 5 | M | B | 132 |
|  |  |  | N/A | N/A | R | Total | No | No | 2.2 | S | R | N/A |
| Yamauchi  et al. | 2018 | 1 | III  (T3a) | 2 | L | Radical | No | No | 6.5 | S | L | 48 |
| Zamarrón  et al. | 2013 | 1 | I  (T1bN0M0) | 2 | L | Total | Mediastinum | No | 0.8 | M (6) | N/A | 120 |

N/A: non-applicable, R: right, L: left, B: both lobes, S: solitary, M: multiple

*AJCC, 8^th^ edition

~ WHO/ISUP or Fuhrman
