# Supplementary material for: Clear Cell Renal Cell Carcinoma Metastasis to the Thyroid: A Narrative Review of the Literature
Source: Cancers (Basel). 2025 Dec 24;18(1):57. doi: 10.3390/cancers18010057 (PMC12785063; doi:10.3390/cancers18010057)
Supplement: Supplementary file 1 [file cancers-18-00057-s001.zip › Table S3.docx]

| Table S3. Analytical presentation of the metastases’ imaging features. | | | |
| --- | --- | --- | --- |
| Author | Year | Cases (n) | Thyroid imaging studies (U/S: ultrasound, EUS: endoscopic ultrasound, CT: computed tomography scan, PET-CT: positron emission tomography,  MRI: magnetic resonance imaging) |
| Abbasii et al. | 2018 | 1 | **CT:** thyroid mass involving both lobes with active bleeding |
| Abdel-Aziz et al. | 2017 | 1 | **EUS:** hypoechoic, round and well-demarcated thyroid mass |
| Abdul-Hadi et al. | 2022 | 1 | **CT:** homogeneous, infiltrative and poorly defined, left anterior neck-thyroid mass with tracheal invasion |
| Al Abdrabalnabi et al. | 2019 | 1 | **U/S:** Left thyroid lobe nodule |
| Albandar et al. | 2017 | 1 | **MRI:** thyroid mass  **PET-CT:** hypermetabolic left thyroid lobe area extending into isthmus |
| Alberto et al. | 2024 | 1 | **U/S:** well-defined, hypoechogenic, ovoid and solid thyroid nodules |
| Alzahrani et al. | 2021 | 1 | **U/S:** four (4) hypervascular thyroid nodules |
| Aydogdu et al. | 2024 | 2 | N/A |
|  |  |  | N/A |
| Babar et al. | 2019 | 1 | **PET-CT**: 17 mm right thyroid nodule |
| Badawi et al. | 2022 | 1 | **U/S:** heterogeneous bilateral thyroid nodules (largest: 19 mm, right lobe) |
| Balta et al. | 2022 | 1 | N/A |
| Bayraktar et al. | 2017 | 1 | **MRI:** midline neck mass extending into the thyroid isthmus  **PET-CT:** nodular lesion in thyroid isthmus |
| Bokhari et al. | 2017 | 1 | **U/S:** three (3) clustered, 6 mm, hypoechoic left thyroid, mid pole nodules  **PET-CT:** hypermetabolic left thyroid, lower pole, 30 mm mass |
| Bruckschen et al. | 2021 | 1 | **CT:** thyroid tumor areas |
| Cesaretti et al. | 2013 | 3 | **U/S:** multiple nodules with a prominent 55 mm left thyroid lobe lesion  **Scintigraphy:** right lobe hot nodule and left lobe cold lesion |
|  |  |  | **U/S:** 7-30mm, bilateral thyroid nodules with increased vascularity  **CT:** cervicomediastinal multinodular goiter dislocating the trachea, the hyoid bone and the laterocervical vascular structures |
|  |  |  | **U/S:** multinodular goiter |
| Chara et al. | 2011 | 1 | **CT:** 40 mm left thyroid lobe nodule  **Scintigraphy:** cold thyroid nodule |
| Chin et al. | 2011 | 1 | N/A |
| Cilengir et al. | 2016 | 1 | **U/S:** macrolobulated, hypoechoic, round-shaped and solid, 30x22 mm thyroid nodule, along with multiple oval-shaped, hypoechoic, solid nodules |
| Citgez et al. | 2011 | 1 | **U/S:** hypoechoic, heterogenous and solid, 34x37 mm nodule in the left thyroid lobe and isthmus and other multiple nodules in both thyroid lobes |
| Connolly | 2018 | 1 | N/A: left thyroid mass with retrosternal extension and tracheal compression |
| D' Angelo et al. | 2014 | 1 | N/A |
| Demir et al. | 2012 | 1 | **U/S:** thyroid lobes enlargement, numerous nodules (one solid at lower right lobe intermixed with the jugular vein along with a thrombus)  **CT:** right lobe mass covering the left lobe |
| Di Furia et al. | 2017 | 1 | **U/S:** 15 mm, solid, hypoechoic, right thyroid lobe nodule with internal vascularization |
| Di Stasi et al. | 2013 | 1 | **U/S:** enlarged thyroid with multiple solid nodules and lymph node involvement  **MRI:** increased thyroid volume, esophageal compression and cervical and submandibular lymph nodes enlargement |
| Falcone et al. | 2018 | 1 | **U/S:** solid and heterogenous nodule with smooth margins |
| Fei et al. | 2023 | 1 | **U/S (Doppler):** hypoechoic left thyroid lobe nodules and VI lymph node group enlargement |
| Foppiani et al. | 2015 | 1 | **U/S:** solid, iso-/hypoechoic left thyroid lobe nodule with internal vascularization  **Scintigraphy:** exclusive tracer uptake in the nodule with inhibition of the surrounding parenchyma |
| García-Trujillo et al. | 2024 | 1 | **U/S:** 25x15 mm right thyroid lobe and 11x 6 mm left thyroid lobe nodules |
| Gawlik et al. | 2023 | 1 | **U/S:** 26x26x20 mm left middle thyroid lobe nodule and two additional mixed cystic, solid and taller than wide nodules with smooth margins  and punctate echogenic foci (TIRADS 4)  **CT:** 26 mm, hypointense left thyroid nodule with coarse calcifications |
| Geisbush et al. | 2019 | 1 | **U/S:** heterogenous, hypoechoic, 33x16x14 mm, lower-mid left lobe pole nodule with central hypervascularity  **PET-CT:** hypermetabolic left thyroid lobe focus |
| Gheorghiu et al. | 2016 | 1 | **U/S:** right thyroid lobe (197.6 mL) mixed with many intranodular cystic areas (TIRADS 4A) and left thyroid lobe (1.8 mL) slightly hypoechoic and heterogenous  with normal vascularization |
| Habibullah et al. | 2020 | 1 | **U/S:** multiple bilateral thyroid nodules (largest: 28 mm, left lobe)  **CT:** thyroid gland enlargement with retrosternal extension, right airway displacement and bilateral heterogenous lesions and some left lobe calcifications |
| Hellums et al. | 2023 | 1 | **U/S:** hypervascular right thyroid lobe mass with suspected thrombosis of the internal jugular vein, CT: 47x35 mm mass within the right thyroid lobe |
| Hryshchyshyn et al. | 2024 | 1 | **U/S:** 15 mm right thyroid lobe nodule and 35 mm left thyroid lobe nodule both hypoechoic with irregular, sharp margins and microcalcification |
| Jackson et al. | 2017 | 7 | N/A |
|  |  |  | N/A |
|  |  |  | N/A |
|  |  |  | N/A |
|  |  |  | N/A |
|  |  |  | N/A |
|  |  |  | N/A |
| Jha et al. | 2016 | 1 | **U/S:** markedly enlarged, heterogenous and hypervascular thyroid along with internal jugular vein thrombosis |
| Jia et al. | 2023 | 3 | **U/S:** heterogenous hypoechoic left lobe nodule (TIRADS 5) and hypoechoic right lobe nodule (TIRADS 4)  **CT:** space occupying thyroid lesion and abnormal IV and V lymph node groups |
|  |  |  | **U/S:** left lobe nodule partially cystic (TIRADS 4) |
|  |  |  | **U/S:** left lobe nodule partially cystic (TIRADS 4) |
| Kaliszewski et al. | 2019 | 9 | N/A |
|  |  |  | N/A |
|  |  |  | N/A |
|  |  |  | N/A |
|  |  |  | N/A |
|  |  |  | N/A |
|  |  |  | N/A |
|  |  |  | N/A |
|  |  |  | N/A |
| Kefeli et al. | 2016 | 1 | **U/S:** 40 mm thyroid mass |
| Khalafi-Nezhad et al. | 2024 | 1 | **U/S:** multiple nodules in both thyroid lobes |
| Khan et al. | 2018 | 1 | **CT:** well-defined and hypodense, 21x28 mm right thyroid lobe nodule |
| Kobayashi et al. | 2015 | 7 | **U/S:** solid, irregular, 36x19x36 mm, right thyroid lobe nodule with internal vascularity |
|  |  |  | **U/S:** solid, irregular, 56x35x48 mm, right thyroid lobe nodule with internal vascularity |
|  |  |  | **U/S:** solid, irregular, 31x19x24 mm, left thyroid lobe nodule with internal vascularity |
|  |  |  | **U/S:** solid, irregular, 39x23x36 mm right thyroid lobe nodule with internal vascularity and thrombi |
|  |  |  | **U/S:** solid, irregular, 81x41x70 mm right thyroid lobe nodule with internal vascularity and thrombi and 27x16x18 left thyroid lobe nodule with internal vascularity |
|  |  |  | **U/S:** solid, irregular, 48x29x30 mm right thyroid lobe nodule with internal vascularity and lymphadenopathy |
|  |  |  | **U/S:** solid, irregular, 48x40x43 mm right thyroid lobe nodule with internal vascularity and thrombi |
| Krishnamurthy et al. | 2014 | 1 | **PET-CT:** ill-defined, heterogenous, right thyroid lobe mass with a large necrotic component and increased metabolic activity |
| Lee et al. | 2011 | 1 | **CT:** 72x55 mm left thyroid lobe and 38x38 mm right thyroid lobe cystic and degenerating masses |
| Lieder et al. | 2017 | 3 | N/A |
|  |  |  | N/A |
|  |  |  | N/A |
| Liu et al. | 2025 | 1 | **U/S:** left thyroid lobe hypoechoic nodule (TIRADS 4a) and right thyroid lobe nodule (TIRADS 3)  **CT:** left thyroid lobe enlargement with poorly defined borders, uneven density and a partially calcified, mass‑like, low‑density, 40x32 mm shadow along with multiple,  small right thyroid lobe nodules |
| Lo et al. | 2015 | 1 | **U/S:** thyroid lobe enlargement with suspicious nodules |
| Macedo-Alves et al. | 2015 | 1 | **U/S:** solid and well-defined heterogenous formation |
| Medas et al. | 2013 | 1 | **U/S:** thyroid enlargement with retrosternal left lobe occupied by an isoechoic vascularized macronodule |
| Moghaddam et al. | 2013 | 1 | N/A |
| Mohammadi et al. | 2014 | 1 | **U/S:** well-defined, hypoechoic, 90x70 mm left thyroid lobe nodule |
| Moradi Tabriz et al. | 2020 | 1 | **U/S:** hypoechoic 38x26 mm and spongiform 24x17 mm right thyroid lobe nodules  **CT:** thyroid enlargement with extension to the thoracic inlet and tracheal compression |
| Nixon et al. | 2011 | 10 | N/A |
|  |  |  | N/A |
|  |  |  | N/A |
|  |  |  | N/A |
|  |  |  | N/A |
|  |  |  | N/A |
|  |  |  | N/A |
|  |  |  | N/A |
|  |  |  | N/A |
|  |  |  | N/A |
| Rahman et al. | 2017 | 1 | N/A |
| Ramírez-Plaza et al. | 2015 | 1 | **U/S:** 22 mm, poorly defined right thyroid lobe nodule within a goiter |
| Ricci et al. | 2021 | 3 | **U/S:** solid, hypoechoic, well-demarcated 22 mm nodule  **PET-CT:** metabolically active right thyroid lobe area |
|  |  |  | **CT:** well-defined, hypodense, right thyroid lobe nodule |
|  |  |  | **U/S:** solid hypoechoic nodule  **PET-CT:** metabolically active right thyroid lobe area |
| Russel et al. | 2016 | 10 | N/A |
|  |  |  | N/A |
|  |  |  | N/A |
|  |  |  | N/A |
|  |  |  | N/A |
|  |  |  | N/A |
|  |  |  | N/A |
|  |  |  | N/A |
|  |  |  | N/A |
|  |  |  | N/A |
| Sarkar et al. | 2024 | 1 | **U/S:** right-sided nodule |
| Sepherd et al. | 2022 | 1 | **U/S:** right sided 57x34x45 mm, hypervascular nodule |
| Shi et al. | 2015 | 1 | N/A |
| Sindoni et al. | 2010 | 1 | **U/S (Doppler):** hypoechoic, 44mm, left thyroid lobe nodule and two (2) 20mm right thyroid lobe nodules with intra-/perinodular blood flow within a goiter |
| Solmaz et al. | 2017 | 1 | **U/S:** thyroid heterogeneity with a hypoechoic, 22 mm, left thyroid lobe nodule  **CT:** thyroid enlargement with a hypoechoic, 22 mm, left thyroid lobe nodule |
| Song et al. | 2017 | 8 | N/A |
|  |  |  | N/A |
|  |  |  | N/A |
|  |  |  | N/A |
|  |  |  | N/A |
|  |  |  | N/A |
|  |  |  | N/A |
|  |  |  | N/A |
| Surov et al. | 2016 | 26 | N/A |
|  |  |  | N/A |
|  |  |  | N/A |
|  |  |  | N/A |
|  |  |  | N/A |
|  |  |  | N/A |
|  |  |  | N/A |
|  |  |  | N/A |
|  |  |  | N/A |
|  |  |  | N/A |
|  |  |  | N/A |
|  |  |  | N/A |
|  |  |  | N/A |
|  |  |  | N/A |
|  |  |  | N/A |
|  |  |  | N/A |
|  |  |  | N/A |
|  |  |  | N/A |
|  |  |  | N/A |
|  |  |  | N/A |
|  |  |  | N/A |
|  |  |  | N/A |
|  |  |  | N/A |
|  |  |  | N/A |
|  |  |  | N/A |
|  |  |  | N/A |
| Tadisina et al. | 2024 | 1 | **U/S:** subcentimeter left thyroid lobe nodules and a 21 mm (TIRADS 5) right inferior thyroid lobe nodule |
| Tang et al. | 2022 | 2 | **U/S:** hypoechoic, circumscribed, solid and cystic 55 mm left thyroid lobe nodule with internal vascularity |
|  |  |  | **U/S:** hypoechoic, solid, 62 mm left thyroid lobe nodule with irregular borders and internal vascularity, suspicious lymph nodes and intratumoral thrombus |
| Tian et al. | 2020 | 1 | **U/S:** right thyroid lobe enlargement with multiple hypoechoic nodules (largest: 56x37 mm) with dotty calcification and irregular fluid sonolucent area  **CT:** 51x43 mm oval lesion with multiple sand-like calcifications |
| Tjahjono et al. | 2021 | 15 | N/A |
|  |  |  | N/A |
|  |  |  | N/A |
|  |  |  | N/A |
|  |  |  | N/A |
|  |  |  | N/A |
|  |  |  | N/A |
|  |  |  | N/A |
|  |  |  | N/A |
|  |  |  | N/A |
|  |  |  | N/A |
|  |  |  | N/A |
|  |  |  | N/A |
|  |  |  | N/A |
|  |  |  | N/A |
| Valdez et al. | 2014 | 1 | **U/S:** 44 mm and 7 mm left thyroid lobe and two (2) 16mm right thyroid lobe nodules |
| Vandermegel | 2021 | 1 | **U/S:** 42x22x28 mm hypoechoic, polylobular, right thyroid lobe nodule with dense central vascularisation (TIRADS 5) |
| Velez Torres et al. | 2022 | 17 | N/A |
|  |  |  | N/A |
|  |  |  | N/A |
|  |  |  | N/A |
|  |  |  | N/A |
|  |  |  | N/A |
|  |  |  | **PET-CT:** N/A |
|  |  |  | **PET-CT:** N/A |
|  |  |  | **PET-CT:** N/A |
|  |  |  | N/A |
|  |  |  | N/A |
|  |  |  | N/A |
|  |  |  | N/A |
|  |  |  | N/A |
|  |  |  | **CT:** N/A |
|  |  |  | N/A |
|  |  |  | N/A |
| Wong et al. | 2017 | 1 | **U/S:** multiple, heterogeneous and hypoechoic nodules in both lobes with solid and cystic areas |
| Xie et al. | 2023 | 1 | **U/S:**  62x21 mm solid and hypoechoic left thyroid gland nodule with irregular shape, poorly defined boundaries, and heterogenous internal echo (TIRADS 4b) along with several anechoic, hypoechoic, and mixed echoic right and left thyroid lobe nodules (TIRADS 3)  **CT:** bilateral thyroid nodules with unclear boundaries |
| Xie et al. | 2023 | 2 | **U/S:** nodule |
|  |  |  | **U/S:** nodule |
| Xu et al. | 2024 | 2 | **U/S:** bilateral thyroid nodules (TIRADS 5) and multiple enlarged neck lymph nodes |
|  |  |  | **U/S:** multiple, mixed echogenic right thyroid lobe nodules (TIRADS 3) with prominent intra-/perinodular blood flow signals |
| Yamauchi et al. | 2018 | 1 | **CT:** 66x58x92 mm left thyroid lobe mass with heterogeneous, strong contrast enhancement and enriched vasculature around the left thyroid lobe |
| Zamarrón et al. | 2013 | 1 | **Scintigraphy:** two (2) cold nodules in a multinodular goiter |
