# Supplementary material for: Clear Cell Renal Cell Carcinoma Metastasis to the Thyroid: A Narrative Review of the Literature
Source: Cancers (Basel). 2025 Dec 24;18(1):57. doi: 10.3390/cancers18010057 (PMC12785063; doi:10.3390/cancers18010057)
Supplement: Supplementary file 1 [file cancers-18-00057-s001.zip › Table S4.docx]

| Table S4. Histopathological features of ccRCC metastases. | | | | | | |
| --- | --- | --- | --- | --- | --- | --- |
| Author | Year | Cases (n) | Metastasis growth pattern | Metastasis cytoplasmic features | Vascular network presence | Metastasis positive immunohistochemistry |
| Abbasii et al. | 2018 | 1 | N/A | N/A | N/A | N/A |
| Abdel-Aziz et al. | 2017 | 1 | Nests | Clear cytoplasm | Yes | CAIX, PAX8 |
| Abdul-Hadi et al. | 2022 | 1 | N/A | N/A | N/A | RCC, CD10, EMA, Vimentin |
| Al Abdrabalnabi et al. | 2019 | 1 | N/A | N/A | N/A | N/A |
| Albandar et al. | 2017 | 1 | N/A | N/A | N/A | CKAE1/AE3, RCC, PAX8, Vimentin |
| Alberto et al. | 2024 | 1 | N/A | Clear cytoplasm | Yes | CK19, CAIX, PAX8, CD10, Vimentin |
| Alzahrani et al. | 2021 | 1 | N/A | N/A | N/A | N/A |
| Aydogdu et al. | 2024 | 2 | N/A | N/A | N/A | N/A |
|  |  |  | N/A | N/A | N/A | N/A |
| Babar et al. | 2019 | 1 | N/A | N/A | N/A | N/A |
| Badawi et al. | 2022 | 1 | Nests and sheets | Clear cytoplasm | Yes | N/A |
| Balta et al. | 2022 | 1 | Tubulocystic | N/A | N/A | CAIX, RCC |
| Bayraktar et al. | 2017 | 1 | Solid, diffuse and glandular | Clear cytoplasm | N/A | PAX8, CD10, EMA |
| Bokhari et al. | 2017 | 1 | Nests | Clear vacuolated cytoplasm | N/A | CAIX, PAX8, EMA |
| Bruckschen et al. | 2021 | 1 | N/A | N/A | N/A | N/A |
| Cesaretti et al. | 2013 | 3 | N/A | N/A | N/A | N/A |
|  |  |  | N/A | N/A | N/A | N/A |
|  |  |  | N/A | Clear | N/A | N/A |
| Chara et al. | 2011 | 1 | N/A | N/A | N/A | N/A |
| Chin et al. | 2011 | 1 | N/A | N/A | N/A | RCC, CD10 |
| Cilengir et al. | 2016 | 1 | N/A | Eosinophilic/clear cytoplasm | N/A | PAX8, EMA, Vimentin |
| Citgez et al. | 2011 | 1 | N/A | N/A | N/A | CD10, EMA |
| Connolly | 2018 | 1 | N/A | N/A | N/A | N/A |
| D' Angelo et al. | 2014 | 1 | Nests and cords | Clear cytoplasm and well marked cytoplasmic membrane | Yes | CD10 |
| Demir et al. | 2012 | 1 | N/A | N/A | N/A | CD10, EMA, Vimentin |
| Di Furia et al. | 2017 | 1 | N/A | N/A | N/A | RCC, CD10, Vimentin |
| Di Stasi et al. | 2013 | 1 | Pseudopapillary and glandular | Clear cytoplasm | Yes | CD10 |
| Falcone et al. | 2018 | 1 | N/A | N/A | N/A | N/A |
| Fei et al. | 2023 | 1 | Nests and sheets | Clear cytoplasm | Yes | RCC, PAX8, CD10 |
| Foppiani et al. | 2015 | 1 | Nests and cords | Clear cytoplasm | N/A | CD10 |
| García-Trujillo et al. | 2024 | 1 | N/A | N/A | N/A | RCC, PAX8, CD10, CAM5.2 |
| Gawlik et al. | 2023 | 1 | N/A | N/A | N/A | PAX8 |
| Geisbush et al. | 2019 | 1 | N/A | N/A | N/A | CD10 |
| Gheorghiu et al. | 2016 | 1 | Alveolar, solid, acinar and microcystic | Clear cytoplasm | N/A | N/A |
| Habibullah et al. | 2020 | 1 | Nests and alveoli | Clear cytoplasm and well marked cytoplasmic membrane | Yes | RCC, EMA, Vimentin |
| Hellums et al. | 2023 | 1 | N/A | N/A | N/A | CAIX, PAX8 |
| Hryshchyshyn et al. | 2024 | 1 | Solid and trabecular | Clear cytoplasm and well marked cytoplasmic membrane | N/A | CAIX, PAX8 |
| Jackson et al. | 2017 | 7 | N/A | N/A | N/A | N/A |
|  |  |  | N/A | N/A | N/A | N/A |
|  |  |  | N/A | N/A | N/A | N/A |
|  |  |  | N/A | N/A | N/A | N/A |
|  |  |  | N/A | N/A | N/A | N/A |
|  |  |  | N/A | N/A | N/A | N/A |
|  |  |  | N/A | N/A | N/A | N/A |
| Jha et al. | 2016 | 1 | N/A | Clear and vacuolated cytoplasm and well marked cytoplasmic membrane | N/A | RCC, CD10 |
| Jia et al. | 2023 | 3 | N/A | N/A | N/A | N/A |
|  |  |  | N/A | N/A | N/A | N/A |
|  |  |  | N/A | N/A | N/A | N/A |
| Kaliszewski et al. | 2019 | 9 | N/A | N/A | N/A | CK7, CD10, Vimentin |
|  |  |  | N/A | N/A | N/A | CK8/18, RCC, CD10 |
|  |  |  | N/A | N/A | N/A | CK7, RCC, CD10, EMA, Vimentin, S-100 protein |
|  |  |  | N/A | N/A | N/A | Vimentin, Thyroglobulin, TTF-1 |
|  |  |  | N/A | N/A | N/A | CK8/18, RCC, CD10 |
|  |  |  | N/A | N/A | N/A | CK7, RCC, CD10, EMA, Vimentin, S-100 protein |
|  |  |  | N/A | N/A | N/A | CKAE1/AE3, CK7, CK19, CD10, Vimentin |
|  |  |  | N/A | N/A | N/A | CK8/18, CD10, Vimentin |
|  |  |  | N/A | N/A | N/A | CK8/18, RCC, CD10 |
| Kefeli et al. | 2016 | 1 | N/A | Clear cytoplasm | Yes | PAX8, CD10, Vimentin |
| Khalafi-Nezhad et al. | 2024 | 1 | N/A | Clear cytoplasm | N/A | CAIX, PAX8, CD10, Vimentin |
| Khan et al. | 2018 | 1 | N/A | N/A | N/A | CAIX, PAX8 |
| Kobayashi et al. | 2015 | 7 | N/A | N/A | N/A | N/A |
|  |  |  | N/A | N/A | N/A | N/A |
|  |  |  | N/A | N/A | N/A | N/A |
|  |  |  | N/A | N/A | N/A | N/A |
|  |  |  | N/A | N/A | N/A | N/A |
|  |  |  | N/A | N/A | N/A | N/A |
|  |  |  | N/A | N/A | N/A | N/A |
| Krishnamurthy et al. | 2014 | 1 | Nests | Clear cytoplasm | Yes | CD10, EMA, Vimentin |
| Lee et al. | 2011 | 1 | Nests | Clear cytoplasm and well marked cytoplasmic membrane | Yes | CKAE1/AE3, CD10, EMA, Vimentin |
| Lieder et al. | 2017 | 3 | N/A | N/A | N/A | N/A |
|  |  |  | N/A | N/A | N/A | N/A |
|  |  |  | N/A | N/A | N/A | N/A |
| Liu et al. | 2025 | 1 | Nests | Clear cytoplasm | Yes | CAIX, PAX8, CD10, Vimentin, MUC-1 |
| Lo et al. | 2015 | 1 | Nests | Clear cytoplasm | Yes | CD10 |
| Macedo-Alves et al. | 2015 | 1 | N/A | Clear cytoplasm | N/A | CD10, Vimentin |
| Medas et al. | 2013 | 1 | N/A | Clear cytoplasm | N/A | CD10 |
| Moghaddam et al. | 2013 | 1 | Lobules | Clear cytoplasm | Yes | CKAE1/AE3, Vimentin |
| Mohammadi et al. | 2014 | 1 | N/A | Granular cytoplasm | N/A | N/A |
| Moradi Tabriz et al. | 2020 | 1 | N/A | N/A | N/A | PAX8, CD10, Vimentin |
| Nixon et al. | 2011 | 10 | N/A | N/A | N/A | N/A |
|  |  |  | N/A | N/A | N/A | N/A |
|  |  |  | N/A | N/A | N/A | N/A |
|  |  |  | N/A | N/A | N/A | N/A |
|  |  |  | N/A | N/A | N/A | N/A |
|  |  |  | N/A | N/A | N/A | N/A |
|  |  |  | N/A | N/A | N/A | N/A |
|  |  |  | N/A | N/A | N/A | N/A |
|  |  |  | N/A | N/A | N/A | N/A |
|  |  |  | N/A | N/A | N/A | N/A |
| Rahman et al. | 2017 | 1 | N/A | Clear cytoplasm | N/A | RCC, CD10, PAX2 |
| Ramírez-Plaza et al. | 2015 | 1 | Nests and cords | Clear cytoplasm | N/A | CD10 |
| Ricci et al. | 2021 | 3 | N/A | N/A | N/A | N/A |
|  |  |  | N/A | N/A | N/A | N/A |
|  |  |  | N/A | N/A | N/A | N/A |
| Russel et al. | 2016 | 10 | N/A | N/A | N/A | N/A |
|  |  |  | N/A | N/A | N/A | N/A |
|  |  |  | N/A | N/A | N/A | N/A |
|  |  |  | N/A | N/A | N/A | N/A |
|  |  |  | N/A | N/A | N/A | N/A |
|  |  |  | N/A | N/A | N/A | N/A |
|  |  |  | N/A | N/A | N/A | N/A |
|  |  |  | N/A | N/A | N/A | N/A |
|  |  |  | N/A | N/A | N/A | N/A |
|  |  |  | N/A | N/A | N/A | N/A |
| Sarkar et al. | 2024 | 1 | N/A | N/A | N/A | PAX8, CD10, Vimentin |
| Sepherd et al. | 2022 | 1 | Nodule | Clear cytoplasm | Yes | CKAE1/AE3, RCC, PAX8 |
| Shi et al. | 2015 | 1 | N/A | N/A | N/A | RCC, CD10, Vimentin, Galectin-3 |
| Sindoni et al. | 2010 | 1 | Ssheets and cords | Clear, slightly granular and pale eosinophilic cytoplasm | Yes | Low-molecular-weight cytokeratin, EMA, Vimentin |
| Solmaz et al. | 2017 | 1 | N/A | Clear cytoplasm | N/A | RCC, CD10, Vimentin |
| Song et al. | 2017 | 8 | N/A | N/A | N/A | N/A |
|  |  |  | N/A | N/A | N/A | N/A |
|  |  |  | N/A | N/A | N/A | N/A |
|  |  |  | N/A | N/A | N/A | N/A |
|  |  |  | N/A | N/A | N/A | N/A |
|  |  |  | N/A | N/A | N/A | N/A |
|  |  |  | N/A | N/A | N/A | N/A |
|  |  |  | N/A | N/A | N/A | N/A |
| Surov et al. | 2016 | 26 | N/A | N/A | N/A | N/A |
|  |  |  | N/A | N/A | N/A | N/A |
|  |  |  | N/A | N/A | N/A | N/A |
|  |  |  | N/A | N/A | N/A | N/A |
|  |  |  | N/A | N/A | N/A | N/A |
|  |  |  | N/A | N/A | N/A | N/A |
|  |  |  | N/A | N/A | N/A | N/A |
|  |  |  | N/A | N/A | N/A | N/A |
|  |  |  | N/A | N/A | N/A | N/A |
|  |  |  | N/A | N/A | N/A | N/A |
|  |  |  | N/A | N/A | N/A | N/A |
|  |  |  | N/A | N/A | N/A | N/A |
|  |  |  | N/A | N/A | N/A | N/A |
|  |  |  | N/A | N/A | N/A | N/A |
|  |  |  | N/A | N/A | N/A | N/A |
|  |  |  | N/A | N/A | N/A | N/A |
|  |  |  | N/A | N/A | N/A | N/A |
|  |  |  | N/A | N/A | N/A | N/A |
|  |  |  | N/A | N/A | N/A | N/A |
|  |  |  | N/A | N/A | N/A | N/A |
|  |  |  | N/A | N/A | N/A | N/A |
|  |  |  | N/A | N/A | N/A | N/A |
|  |  |  | N/A | N/A | N/A | N/A |
|  |  |  | N/A | N/A | N/A | N/A |
|  |  |  | N/A | N/A | N/A | N/A |
|  |  |  | N/A | N/A | N/A | N/A |
| Tadisina et al. | 2024 | 1 | Nests | Clear cytoplasm | N/A | PAX8, CD10 |
| Tang et al. | 2022 | 2 | N/A | Large vacuolated clear/eosinophilic cytoplasm | Yes | N/A |
|  |  |  | N/A | N/A | N/A | N/A |
| Tian et al. | 2020 | 1 | N/A | N/A | N/A | CKAE1/AE3, CK8/18, CD10, Vimentin |
| Tjahjono et al. | 2021 | 15 | N/A | N/A | N/A | N/A |
|  |  |  | N/A | N/A | N/A | N/A |
|  |  |  | N/A | N/A | N/A | N/A |
|  |  |  | N/A | N/A | N/A | N/A |
|  |  |  | N/A | N/A | N/A | N/A |
|  |  |  | N/A | N/A | N/A | N/A |
|  |  |  | N/A | N/A | N/A | N/A |
|  |  |  | N/A | N/A | N/A | N/A |
|  |  |  | N/A | N/A | N/A | N/A |
|  |  |  | N/A | N/A | N/A | N/A |
|  |  |  | N/A | N/A | N/A | N/A |
|  |  |  | N/A | N/A | N/A | N/A |
|  |  |  | N/A | N/A | N/A | N/A |
|  |  |  | N/A | N/A | N/A | N/A |
|  |  |  | N/A | N/A | N/A | N/A |
| Valdez et al. | 2014 | 1 | Nests | Clear cytoplasm | Yes | RCC, CD10 |
| Vandermegel | 2021 | 1 | N/A | Clear cytoplasm | N/A | CK19, PAX8, CD10,Vimentin |
| Velez Torres et al. | 2022 | 17 | N/A | N/A | N/A | N/A |
|  |  |  | N/A | N/A | N/A | N/A |
|  |  |  | N/A | N/A | N/A | N/A |
|  |  |  | N/A | N/A | N/A | N/A |
|  |  |  | N/A | N/A | N/A | N/A |
|  |  |  | N/A | N/A | N/A | N/A |
|  |  |  | N/A | N/A | N/A | N/A |
|  |  |  | N/A | N/A | N/A | N/A |
|  |  |  | N/A | N/A | N/A | N/A |
|  |  |  | N/A | N/A | N/A | N/A |
|  |  |  | N/A | N/A | N/A | N/A |
|  |  |  | N/A | N/A | N/A | N/A |
|  |  |  | N/A | N/A | N/A | N/A |
|  |  |  | N/A | N/A | N/A | N/A |
|  |  |  | N/A | N/A | N/A | N/A |
|  |  |  | N/A | N/A | N/A | N/A |
|  |  |  | N/A | N/A | N/A | N/A |
| Wong et al. | 2017 | 1 | Nests | Abundant glycogen‑rich cytoplasm | Yes | RCC, CD10 |
| Xie et al. | 2023 | 1 | N/A | N/A | N/A | CAIX, PAX8, CD10, Vimentin, Galectin-3 |
| Xie et al. | 2023 | 2 | N/A | N/A | N/A | N/A |
|  |  |  | N/A | N/A | N/A | N/A |
| Xu et al. | 2024 | 2 | Nests and sheets | N/A | N/A | CK19, PAX8, CD10, Cyclin D1 |
|  |  |  | N/A | N/A | N/A | CKAE1/AE3, PAX8, CD10, P504S, Cyclin D1 |
| Yamauchi et al. | 2018 | 1 | N/A | Clear/eosinophilic cytoplasm | N/A | CD10, Vimentin |
| Zamarrón et al. | 2013 | 1 | N/A | N/A | N/A | CKAE1/AE3, CD10, EGFR, VEGFR2 |
