# Supplementary material for: Clear Cell Renal Cell Carcinoma Metastasis to the Thyroid: A Narrative Review of the Literature
Source: Cancers (Basel). 2025 Dec 24;18(1):57. doi: 10.3390/cancers18010057 (PMC12785063; doi:10.3390/cancers18010057)
Supplement: Supplementary file 1 [file cancers-18-00057-s001.zip › Table S5.docx]

| **Table S5.** Results for the evaluation of study characteristics in the OS (from initial diagnosis). | | | |
| --- | --- | --- | --- |
| **Characteristic** | **HR (95% CI)** | **p-value (logrank)** | **N** |
| Gender | 1.18 (0.49-2.81) | 0.7120 | 138 |
| Other synchronous neoplasia | 2.17 (0.39-11.93) | 0.3629 | 66 |
| Solitary (S) / Multiple (M) | 2.33 (0.52-10.36) | 0.2547 | 101 |
| Laterality (R: right, L: left, B: both lobes) |  | 0.9217 | 89 |
| Grade (WHO/ISUP or Fuhrman) on diagnosis |  | 0.2353 | 36 |
