# Supplementary material for: Clear Cell Renal Cell Carcinoma Metastasis to the Thyroid: A Narrative Review of the Literature
Source: Cancers (Basel). 2025 Dec 24;18(1):57. doi: 10.3390/cancers18010057 (PMC12785063; doi:10.3390/cancers18010057)
Supplement: Supplementary file 1 [file cancers-18-00057-s001.zip › Table S6.docx]

| Table S6. Results for the evaluation of study characteristics in the OS (from diagnosis of metastasis). | | | |
| --- | --- | --- | --- |
| **Label** | **Measure** | **p-value (log rank)** | **N** |
| Gender | 0.73 (0.22-2.43) | 0.6014 | 47 |
| Other synchronous neoplasia | 1.7 (0.32-9.05) | 0.5309 | 36 |
| Solitary (S) / Multiple (M) | 2.82 (0.28-28.21) | 0.3636 | 31 |
| Laterality (R: right, L: left, B: both lobes) |  | 0.3385 | 40 |
| Grade (WHO/ISUP or Fuhrman) on diagnosis |  | 1.0000 | 17 |
